# Evaluating the efficacy, impact, and feasibility of community-based house screening as a complementary malaria control intervention in southern Africa: a study protocol for a household randomized trial

**DOI:** 10.1186/s13063-021-05768-7

**Published:** 2021-12-06

**Authors:** Onyango P. Sangoro, Ulrike Fillinger, Kochelani Saili, Theresia Estomih Nkya, Rose Marubu, Freddie Masaninga, Sonia Casimiro Trigo, Casper Tarumbwa, Busiku Hamainza, Candrinho Baltazar, Joseph Mberikunashe, Brian Chisanga, Kassie Menale, Emmanuel Chanda, Clifford Maina Mutero

**Affiliations:** 1 International Centre of Insect Physiology and Ecology, Nairobi, Kenya; 2 School of Health Systems & Public Health, University of Pretoria, Pretoria, South Africa; 3 World Health Organization, Lusaka, Zambia; 4 World Health Organization, Maputo, Mozambique; 5 World Health Organization, Harare, Zimbabwe; 6 National Malaria Elimination Centre, Lusaka, Zambia; 7 National Malaria Control Programme, Maputo, Mozambique; 8 National Malaria Control Programme, Harare, Zimbabwe; 9 Department of Social Sciences, Wageningen University and Research, Wageningen, Netherlands; 10 World Health Organization, Regional Office for Africa, Brazzaville, Congo

**Keywords:** Integrated vector management, House screening, Malaria elimination, Residual malaria transmission

## Abstract

**Background:**

Concerted effort to control malaria has had a substantial impact on the transmission of the disease in the past two decades. In areas where reduced malaria transmission is being sustained through insecticide-based vector control interventions, primarily long-lasting insecticidal nets (LLINs) and indoor residual spraying (IRS), non-insecticidal complementary tools will likely be needed to push towards malaria elimination. Once interruption in local disease transmission is achieved, insecticide-based measures can be scaled down gradually and eventually phased out, saving on costs of sustaining control programs and mitigating any unintended negative health and environmental impacts posed by insecticides. These non-insecticidal methods could eventually replace insecticidal methods of vector control.

House screening, a non-insecticidal method, has a long history in malaria control, but is still not widely adopted in sub-Saharan Africa. This study aims to add to the evidence base for this intervention in low transmission settings by assessing the efficacy, impact, and feasibility of house screening in areas where LLINs are conventionally used for malaria control.

**Methods:**

A two-armed, household randomized clinical trial will be conducted in Mozambique, Zambia, and Zimbabwe to evaluate whether combined the use of house screens and LLINs affords better protection against clinical malaria in children between 6 months and 13 years compared to the sole use of LLINs. Eight hundred households will be enrolled in each study area, where 400 households will be randomly assigned the intervention, house screening, and LLINs while the control households will be provided with LLINs only. Clinical malaria incidence will be estimated by actively following up one child from each household for 6 months over the malaria transmission season. Cross-sectional parasite prevalence will be estimated by testing all participating children for malaria parasites at the beginning and end of each transmission season using rapid diagnostic tests.

CDC light traps and pyrethrum spray catches (PSC) will be used to sample adult mosquitoes and evaluate the impact of house screening on indoor mosquito density, species distribution, and sporozoite rates.

**Discussion:**

This study will contribute epidemiological data on the impact of house screening on malaria transmission and assess the feasibility of its implementation on a programmatic scale.

**Trial registration:**

ClinicalTrials.gov PACTR202008524310568. Registered on August 11, 2020.

**Supplementary Information:**

The online version contains supplementary material available at 10.1186/s13063-021-05768-7.

## Administrative information

Note: the numbers in curly brackets in this protocol refer to SPIRIT checklist item numbers. The order of the items has been modified to group similar items (see http://www.equator-network.org/reporting-guidelines/spirit-2727-statement-defining-standard-protocol-items-for-clinical-trials/). above the administrative information table.

**Table:** 

Title {1}	Evaluating the efficacy, impact, and feasibility of community-based house screening as a complementary malaria control intervention in southern Africa: a study protocol for a household randomized trial.
Trial registration {2a and 2b}.	Pan African Clinical Trial Registry. Registration number PACTR202008524310568.
Protocol version {3}	Version 7
Funding {4}	World Health Organization Regional Office for Africa (WHO AFRO), - Financial and technical support. United Nation Environment Programme - Financial support. Global Environment Facility - Financial support.
Author details {5a}	1. International Centre of Insect Physiology and Ecology, Nairobi, Kenya 2. World Health Organization, Lusaka, Zambia 3. World Health Organization, Maputo, Mozambique 4. World Health Organization, Harare, Zimbabwe 5. National Malaria Elimination Centre, Lusaka, Zambia 6. National Malaria Control Programme, Maputo, Mozambique 7. National Malaria Control Programme, Harare, Zimbabwe 8. World Health Organization, Regional Office for Africa, Brazzaville, Congo 9. Department of Social Sciences, Wageningen University and Research, Wageningen, Netherlands 10. School of Health Systems & Public Health, University of Pretoria, Pretoria, South Africa.
Name and contact information for the trial sponsor {5b}	World Health Organization Regional Office for Africa (WHO-AFRO), P.O. Box 06 Djoue, Congo Brazzaville
Role of sponsor {5c}	World Health Organization Regional Office for Africa (WHO-AFRO), participated in the study design, monitoring of the study implementation and will also be involved in reviewing manuscripts submitted to peer-reviewed journals for publication.

## Introduction

### Background and rationale {6a}

Insecticide-based malaria control tools have led to significant reductions in malaria transmission, morbidity, and mortality in the past two decades [1]. However, recent reports indicate that progress in malaria control has stalled [2, 3] mainly due to the reallocation of funds previously available for malaria control, lack of political commitment to invest in malaria control programs and defined limits of current vector control strategies. That lack of adequate funding has resulted in the weakening of malaria control programs and the attendant coverage gaps of malaria interventions. One of the pillars of the Global Technical Strategy (GTS) [4] to reduce malaria morbidity and mortality by 90% globally is to ensure universal access to malaria prevention interventions [4] at an annually recurrent cost of between $6.4 and $8.7 billion from 2020 to 2030 [4]. In 2018, only $2.7 billion was invested in malaria control, about one third of the amount needed to attain the 2020 GTS targets [3]. Given the current COVID-19 pandemic [5] and its dire economic consequences worldwide, it is unlikely for this funding gap to be met any time soon. Consequently, the GTS goal of 90% coverage with currently supported malaria interventions will fall short, leaving a significant proportion of the at-risk population unprotected. This coverage gap may lead to a resurgence of malaria in regions where it had been previously been controlled and might jeopardize the elimination goal, especially in countries at the fringe of malaria transmission, like the Southern African Region [6]. Mathematical models estimate significant funding is needed to achieve the GTS strategy [4]. However, the return of investment of elimination (up to $300 billion) far outweighs the input costs, not to mention other costs saved from reduced malaria treatment and increase in the labor force from reduced illness [7]. Given the status of the world economy, it is unlikely that these funds will be available [8]. It is therefore prudent that financing mechanisms for malaria control are reviewed, adjusted, and diversified to fit the endemic countries that rely on international funding [9] and sustainable novel strategies for malaria control are developed for these settings [10].

In 2001, leaders from African Union countries pledged to commit 15% of their national budget to improve the health of their countries [11]. Two decades later, these commitments are yet to be honored [12]. This lack of political will by African governments, where the malaria burden is highest, further widens the coverage gap of malaria interventions and escalates the threat to progress made in malaria control. Consequently, new strategies are being developed to keep malaria control and elimination high on the developmental and political agenda and sustain financing of control programs to achieve the 2030 GTS targets [13]. These strategies include developing multisectoral partnerships with sectors such as housing and agriculture to tackle malaria transmission, regional initiatives such as the Eliminate-8 (E-8) initiative for cross-border surveillance of malaria, expanding the domestic financing base and strengthening the health systems of endemic countries [4]. In 2018, the WHO launched the High burden High Impact initiative (HBHI) which advocated for the development of country tailored goals and strategies towards malaria control and elimination. This initiative also emphasized the translation of stated political commitments into resources and tangible actions [9].

Malaria transmission is inter-linked with socio-demographic and environmental factors [7]. Socio-economic development is likely to improve housing, nutrition, access to health care, and education which all result in improved health and reduce malaria transmission. Environmental factors such as climate change and land use will likely change the geographic distribution of malaria and shift malaria transmission upwards or downwards. Population growth and movement into urban areas is will reduce malaria transmission because of improved standards of living, access to health care, and destruction of mosquito breeding sites [7]. All these factors, coined, megatrends [14], will lead to reduced malaria transmission overall, yet are unlikely to lead to elimination unless the tracks are laid down now to diversify interventions and to take advantage of some of the changes [7].

The WHO and the international malaria community have recommended the implementation of integrated vector management (IVM) strategy to sustainably control, and ultimately, eliminate malaria [15] and especially emphasized the need for local action [9]. IVM underscores the need for multi-sectoral collaboration and action, evidence-based decision-making, social mobilization, and utilization of existing systems and locally available resources [16].

Most malaria transmission in sub-Saharan Africa still occurs indoors despite increased vector control efforts with intra-domiciliary measures, LLINs, and IRS [17], with reports estimating human exposure to mosquito bites to be greater indoors than outdoors [18–20]. This occurrence highlights the need for the development of tools that can attack this exposure to mosquitoes indoors despite the extensive employment of LLINs and IRS.

Historically, house screening studies have demonstrated a reduction in mosquito numbers indoors and subsequently reduced the risk of malaria transmission [21, 22]. However, these studies did not generate robust evidence for house screening [22]. House screening works by physically deterring the entry of mosquitoes into houses, thereby reducing human exposure to infectious bites indoors [23, 24]. In addition, the use of non-insecticidal house screens presents an opportunity for minimizing reliance on insecticide-based measures for malaria control. This strategy could supplement the primary insecticide-based tools in settings where there is an extensive spread of insecticide resistance as it would target all vectors, thereby having a greater impact on malaria transmission than the sole use of insecticide-based measures which only targets susceptible mosquitoes. Gradually, as malaria gets controlled and eliminated this might become a dominant tool to maintain the success.

Despite its potential, house screening is yet to be embedded into the malaria control policy as a vector control tool. This is because with the advent of DDT in the 1950s, malaria control shifted focus to insecticide-based interventions and as a result, limited evidence on the epidemiological impact of environmental management, including house screening, was generated [22].

To date, only two randomized controlled trials have been conducted to evaluate the impact of house screening against malaria transmission [25, 26]. Another house screening trial is currently being implemented in the Gambia [27]. However, there is a need for extensive data from different eco-epidemiological settings in order to incorporate house improvement into an integrated program for malaria control. Consequently, the World Health Organization-Regional Office for Africa (WHO-AFRO) and the International Centre of Insect Physiology and Ecology (*icipe*) are conducting a multi-country study in southern Africa on the additional impact of house screening on malaria transmission and clinical disease outcomes to support the formulation of policy guidelines around house improvement for malaria control. This study is being implemented by the Ministries of Health in Mozambique, Zambia, and Zimbabwe, through their respective National Malaria Control Programmes (NMCP) with technical assistance from WHO-AFRO and *icipe*.

House screening presents a pragmatic tool for malaria control as housing conditions are improving in sub-Saharan Africa, and malaria control programs can tap into this opportunity to develop housing that protects against malaria transmission [22]. In addition, improved housing might offer an avenue for sustainable malaria control as it will be cheaper compared to LLINs and IRS and may replace these insecticide-based measures in the long run.

### Objectives {7}

The overall aim of the study is to assess the efficacy, impact, and feasibility of house screening as an additional anti-malaria intervention in areas where LLINs are conventionally used for malaria control in Zambia, Zimbabwe, and Mozambique.

#### Clinical outcomes

##### Primary objective

To evaluate whether house screening of all mosquito entry points (in semi-modern houses) in addition to LLIN use provides greater protection against clinical malaria in children between 6 months and 13 years than the sole use of LLINs.

##### Secondary objective

To evaluate the impact of house screening and LLIN use on malaria parasite prevalence in children (6 months–13 years) in the intervention compared to the control cohort of the study.

#### Entomological outcomes

##### Primary objective

To evaluate the impact of house screening and LLINs on mosquito host-seeking and indoor resting densities.

##### Secondary objective

To evaluate the impact of house screening and LLINs on the entomological inoculation rate.

#### Socio-economic outcomes

##### Primary objective

To assess the incremental costs of house screening as a supplementary mosquito abatement tool.

##### Secondary objectives

To explore the acceptability of house screening as a malaria control tool in the community.

#### Trial design {8}

This study is a multicounty, two-armed household randomized controlled trial using a generalized randomized block design, with the village as the block. Prior to the selection of the study households in each of the three study locations in the three countries, a baseline household census will be conducted in each village in the target areas. A database of study households that meet the eligibility criteria will then be generated.

During the household census, household access to LLINs will be assessed to ensure that there is an LLIN for every sleeping place in the enumerated household, assuming two people use a single net. House type and potential mosquito entry points for each enumerated household will also be recorded. If the household does not have an LLIN for every sleeping space, this will be issued during the baseline household socio-demographic survey that will be conducted after the household census. The socio-demographic survey will be conducted in 800 randomly selected study households. After the baseline socio-demographic survey, one child between 6 months and 13 years will be randomly selected from the 800 study households to be included in the study cohort. This study cohort will then be screened for malaria parasites (parasitological survey) at the beginning and end of the transmission season. Sentinel households, 120, will be selected from the enrolled study households for an entomological sampling of adult mosquitoes. Exposure to indoor mosquito bites will be estimated by routine surveillance of the sentinel households using CDC light traps and while indoor mosquito resting densities will be estimated using Pyrethrum Spray Catch (PSC) technique. Three months prior to the start of the transmission season (January-May), 400 enrolled households will be randomized to the intervention arm and all mosquito entry points (windows, eaves and doors) screened.

The remaining 400 households will be assigned to the control (no-screening) arm. Households lost to follow-up will be replaced with non-randomized households enumerated during the household census.

As malaria risk may vary between villages, the number of intervention and control households per village will be balanced. A maximum of 10% of all households per village will be enrolled for house screening to avoid the potential diversion of mosquitoes from screened to non-screened households. A similar number of households will be assigned to the control arm in the same village so that the number of intervention and control households will be evenly distributed in each village across the study sites.

**Fig. 1:**
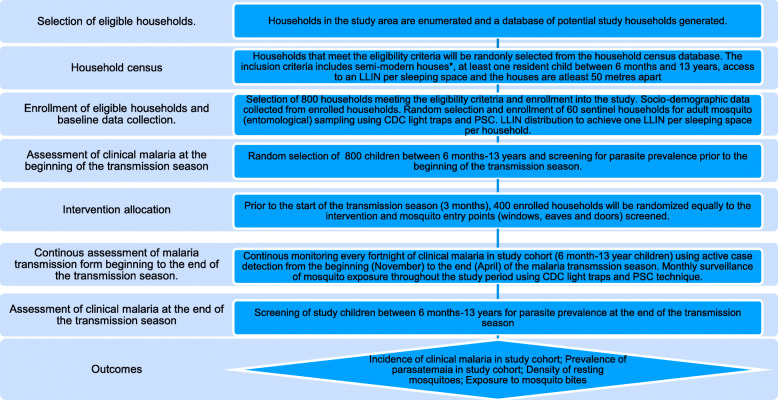
Schematic illustration of trial design

The main study outcome will be clinical malaria incidence monitored in the study children cohort by actively following each child enrolled in the study (active case detection-ACD), measuring their body temperature every fortnight for a period of 3–6 months during the high malaria transmission season (January to May) for 2 subsequent transmission seasons of the study. Children presenting with a fever above 37°C or reporting febrile illness in the past week will be tested for malaria using an RDT. The secondary outcome will be parasite prevalence assessed by screening study children at the beginning and end of each transmission season for the 2 consecutive transmission seasons of the study. Vector biting and resting densities indoors will be monitored throughout the study period using CDC light traps and PSC techniques in intervention and control sentinel houses. Acceptability of the proposed intervention, cost of house screening vis a vis LLINs, and the feasibility of scale-up to the community will be assessed using focus group discussions (FGDs) and key informant interviews (KII) with project stakeholders (Fig. 1).

## Methods: participants, interventions, and outcomes

### Study setting {9}

The study will be conducted in three southern African countries: Zambia, Zimbabwe, and Mozambique. Study areas were selected in malaria transmission hotspots where the use of LLINs is promoted by the Ministry of Health, but IRS is not routinely implemented. The project countries had similar profiles in terms of malaria incidence/prevalence, primary malaria vectors, parasites transmitting malaria, vector resistance to insecticides, tools employed in malaria control, and transmission season (Table 1).

**Table 1:** Malaria epidemiology in the project countries

Countries	Zambia	Mozambique	Zimbabwe	References
Malaria prevalence/incidence	Prevalence 9% in 2018 for under 5’s.	Weighted prevalence of 38.9% in 2018	Incidence 20.5/1000 population in 2016	[28–30]
Primary vectors	*An. funestus* *An. gambiae* s.s *An. arabiensis* [31]	*An. gambiae* s.l. *An. funestus* s.l.	*An. gambiae* s.l. *An. funestus* s.l.	[28, 32, 33]
Secondary vectors	*An. coustani, An. squamosus, An. pretoriensis* *An. rufipes*	*An. coustani, An. tenebrosus, An. ziemanni.*	*An. coustani, An. natalensis, An. pretoriensis*	[34–38]
Primary parasite prevalence	*P. falciparum* (95%) *P. ovale* (2%) *P. malariae* (3%)	*P. falciparum* (90%) *P.* *malariae* (9%) *P. ovale* (1%)	*P. falciparum* (98%) *P. ovale* & *P. malariae* (2%)	[28, 29, 33]
Insecticide resistance	Both *An. funestus* and *An. gambiae* s.s. resistant to deltamethrin, lambda-cyhalothrin, permethrin and DDT.	Both *An. funestus* s.l. and *An. gambiae* s.l. resistant to deltamethrin, alphacypermethrin, lambda-cyhalothrin, permethrin and DDT in some areas of Mozambique.	*An. arabiensis* reported to be resistant to DDT and permethrin. *An. funestus* was reported to be resistant to carbamates and pyrethroids. *An. gambiae* s.l. reported to be resistant to bendiocarb and lambda-cyhalothrin.	[29, 33, 39]
Malaria control tools	IRS, LLINs, IPT_P_, RDTs, and case management using ACTs [40].	IRS, LLINs, RDTs, case management using ACTs, Social & Behaviour Communication Change (SBCC) and entomological monitoring and surveillance.	IRS, LLINs, RDTs, case management using ACTs, therapeutic efficacy testing (TET), Community-based management of malaria, IPT_P_, and entomological surveillance.	[29, 33, 40]
Transmission season	January–April	January–April	January–April	[28, 30, 33]

### Nyimba, Zambia

**Fig. 2:**
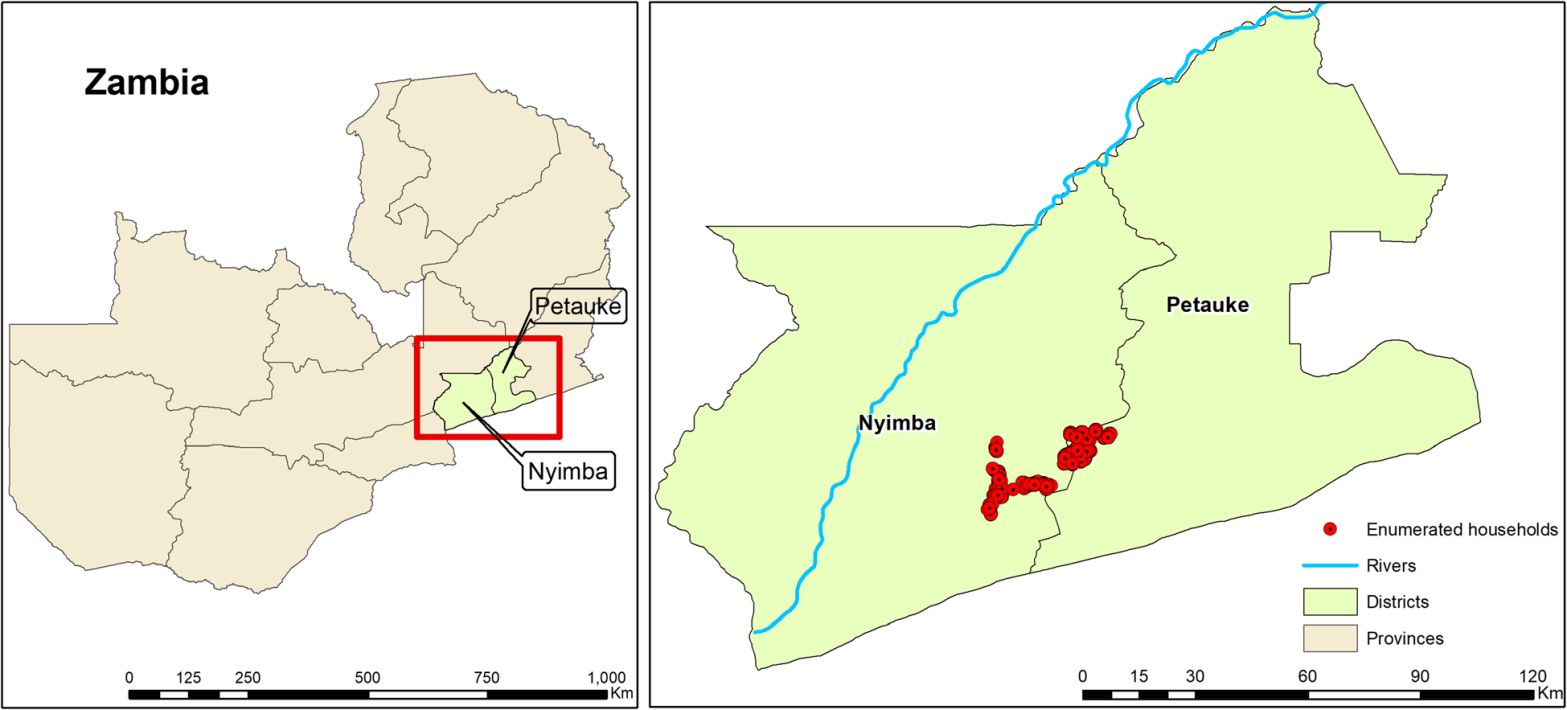
Map showing the study area in Nyimba District, Eastern Province, Zambia

The house screening trial will be conducted in the Nyimba District located 350 km East of Lusaka in Eastern Province, Zambia. Nyimba has a cool dry winter from May to August and a warm wet season from December to April, which is the main malaria transmission season [41]. *Plasmodium falciparum* in Nyimba is vectored by *An. funestus* which mediates an entomological inoculation rate (EIR) of 70 infectious bites per unprotected user of LLIN [42]. The average annual temperature is 25°C, and the annual average rainfall was 175 mm from 2009 to 2020 [43] (Fig. 2).

### Chiredzi, Zimbabwe

**Fig. 3:**
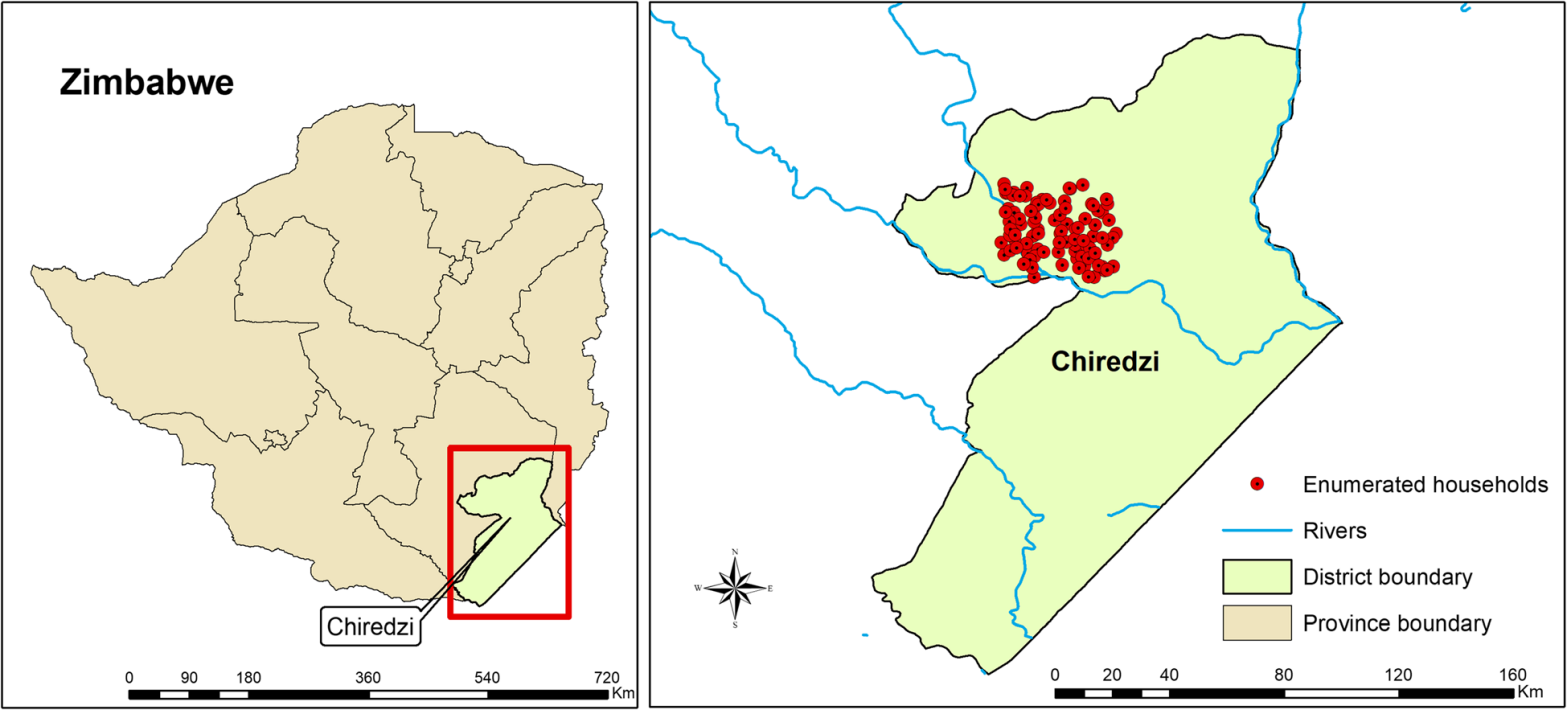
Map showing the study area in Chiredzi District, Masvingo Province, Zimbabwe

The evaluation of house-screening will be explored in Chiredzi District located in Masvingo province in South-Eastern Zimbabwe situated at 21° 02′ 20′′ S 31° 40′ 40′′ E. Chiredzi district receives on average 78 mm of annual rainfall and has an annual average temperature of 26°C [43]. The main malaria vector found in this area is *An. funestus. An. quadriannulatus* was found to be the most predominant of the *An. gambiae.* s.l. complex. Other anophelines present in the area were *An*. *pretoriensis*, *An*. *squamous*, *An*. *rufipes*, *An*. *coustani*, and *An*. *pharoensis*. The major control measures employed in the area are IRS, LLINs, larval source management (LSM), and prompt case management [44] (Fig. 3).

### Chokwe, Mozambique

**Fig. 4:**
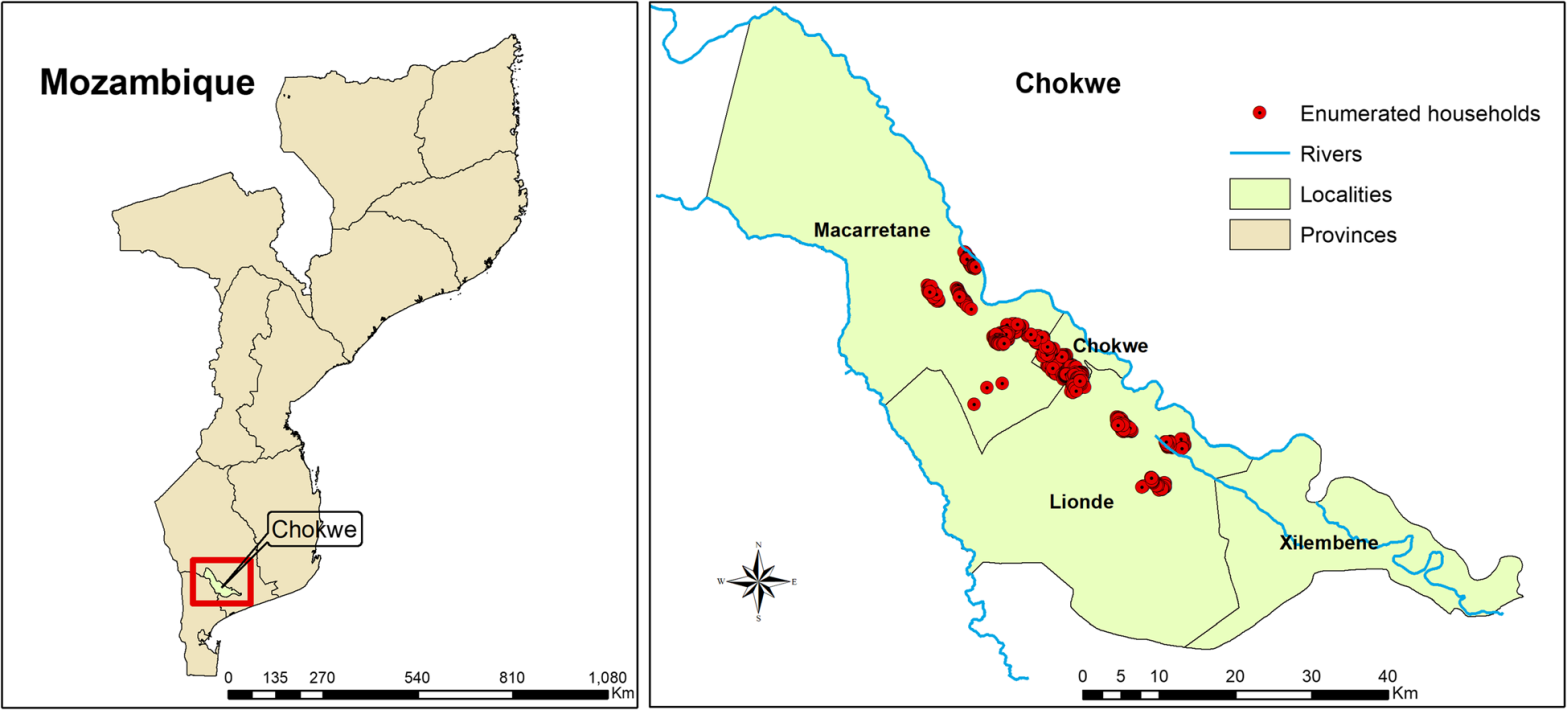
Map showing study areas in Chokwe District, Mozambique (HDSS study area), in Chokwe District, Gaza Province, Mozambique

The house-screening trial will be conducted in Chokwe District, Gaza Province, specifically within the study area of the Chokwe Health Demographic Surveillance System (CHDDS), at 24° 33′ 37′′ S, 33° 1′ 20′′ E in southern Mozambique. Chokwe district receives on average 72 mm of annual rainfall and has an annual average temperature of 26°C. The major *P. falciparum* vectors are *An. funestus* and *An. gambiae* s.l. *An. funestus* are recorded to be highly resistant to pyrethroids in Chokwe [45] and as a result, IRS using DDT has been used for malaria control in this area since 2006 [45] (Fig. 4).

### Eligibility criteria {10}

The households to be enrolled in the study will be assigned the following inclusion criteria:

- Houses that have either a tin or tiled roof; mud, stone, or wooden walls and earthen or cemented floors (*semi-modern) with few open spaces for potential mosquito entry and screening; the houses should not be in such a debilitated state that it is impossible to cover up the mosquito entry points.
- Houses are at least 50 m apart to prevent the diversion of mosquitoes from the treatment to control houses.
- Houses have at least 1 resident child between 6 months and 13 years of age.
- The resident child recruited into the study cohort will stay in the study area for the whole study period and will not be allowed to travel out of the study area during the project period.
- Houses in which the household head/guardian has signed the study informed consent.

### Who will take informed consent? {26a}

Once all houses (800) to be enrolled in the study have been identified, consent will be sought from the household heads/guardians for a resident child between 6 months and 13 years, to join the study cohort for the malaria incidence (clinical malaria) and prevalence (parasite density) surveys.

Written and verbal information will be provided to household heads/guardians on the objectives, benefits, and potential risks of the study using a local language with which they are familiar. The household heads/guardians will be advised that participation is voluntary. Only children whose parents have given written consent for their child to be included in the study will be enrolled.

The participant child will be selected randomly among those aged 6 months to 13 years from the 800 houses enrolled. During the blood specimen collection for clinical malaria infection and parasite prevalence, assent will be sought from children above 8 years, and if the assent is denied, this child will be excluded from data collection. No distinctions will be made regarding the gender or ethnic group of the household.

Consent will also be sought from household heads for their houses to be used as sentinel stations for the sampling of adult mosquitoes using CDC light traps and PSC techniques.

Any child testing positive for malaria parasites will be treated as per respective national malaria diagnosis and treatment guidelines [46].

### Additional consent provisions for collection and use of participant data and biological specimens {26b}

On the actual day of blood specimen collection, the household heads/guardians will be presented with another consent form before a blood specimen is collected from the study children. If consent is obtained, then the field workers will collect the data. However, if consent is denied, blood specimens will not be collected from the child.

#### Interventions

##### Explanation for the choice of comparators {6b}

Long-lasting insecticidal nets (LLINs) will be the comparator in this study. This is because LLINs are the currently recommended intervention by the study countries’ NMCPs and by WHO. Universal coverage of LLINs for personal protection is also the gold standard malaria intervention and the only ethical comparator that can be used when testing new malaria control tools as has been demonstrated to be both effective and readily available. Therefore, we will ensure universal access to LLINs and then evaluate the additional benefits of house screening against malaria transmission.

#### Intervention description {11a}

##### House screening

In the target areas of all three countries, 400 houses will be screened with non-insecticide PVC-coated fiberglass netting by trained artisans recruited from each study village. The installations are expected to take about 2–3 months to complete. House residents will be trained on the care needed to keep the screens intact and avoid activities that could damage the screens. Screened houses will be examined by field assistants during entomological sampling of adult mosquitoes in sentinel households when conducting active case detection of malaria incidence every fortnight during the transmission season and during the cross-sectional parasite density surveys (parasite prevalence). The integrity of the screens will be recorded during these observations. Other routine procedures to reduce mosquito entry into houses, such as shutting the door whenever an occupant enters or leaves the house between 18.00h and 06.00h, will be emphasized and adherence to these practices monitored passively by the study field team during the above activities. These observations will be recorded in structured forms in order to quantify the frequency of their occurrence over time.

##### Criteria for discontinuing or modifying allocated interventions {11b}

Withdrawals may also occur if a study subject has any significant adverse events such increase in upper respiratory tract infections (URTis) in household members in screened households compared to non-screened households.

##### Strategies to improve adherence to interventions {11c}

Household heads and residents will be trained on the care needed to keep the screens effective, such as avoiding activities that may poke holes onto the nets or cause damages that will create spaces that would allow mosquito entry into the houses. Other routine procedures that reduce mosquito entry into houses such as closing windows and doors early will be emphasized and adherence to this practice monitored passively by the study team during entomological, clinical, and parasitological data collection as described above. The community members in the study areas will also be continually engaged on the benefits of participating in the study and the importance of following the recommended procedures above through community meetings to encourage adherence to malaria and parasitological surveys.

##### Relevant concomitant care permitted or prohibited during the trial {11d}

As this study aims to assess the additional impact of house screenings to LLINs in preventing malaria transmission, the use of IRS in the study target area will be prohibited.

##### Provisions for post-trial care {30}

At the end of the study, households in the control arm will be given the option of screening their households at project cost. Also, households in the intervention arm will be given the option of having the screens removed from their houses if they so wish.

#### Outcomes {12}

##### *Clinical endpoints*

The primary endpoint will be the incidence of clinical malaria in children between 6 months and 13 years. Clinical malaria will be assessed by active case detection over the main transmission season (January-April). The axillary temperature will be taken every fortnight by CHWs from all enrolled study children and if ≥ 37.5°C or history of fever in the past 48 h is reported, the child will be tested for malaria using a rapid diagnostic test kit (RDT). Blood specimens will also be collected using thick and thin blood slide smears for parasite identification and a dry blood spot (DBS) using a filter paper for later malaria analysis using PCR techniques.

The secondary endpoint will be the prevalence of malaria parasitemia in the study children, and the average parasite density in each study arm will be determined. Cross-sectional surveys will be done before (November) and after the main transmission season (May). A finger prick sample will be taken for an RDT and a thick and thin blood smear and DBS prepared for microscopy and molecular analysis in the laboratory.

RDT positive children will be provided with a full dose of *Artemether-lumefantrine* (AL) for treatment by a trained health worker following in-country national guidelines [47].

##### *Entomological endpoints*

The primary entomological endpoint will be the relative indoor mosquito density, expressed as the average number of mosquitoes caught per trap night in each study arm. The secondary endpoint will be the entomological inoculation rate (EIR) incorporating the blood meal source (human biting rate) and the mosquito sporozoite positivity per study arm.

A total of 30 sentinel houses per study arm (*N*=60) per country will be randomly selected for adult mosquito collections. The household selection will be stratified by village and geographical area so that an equal number of sentinel houses are selected in each village and equally spread over the study area. This stratified randomization by the village will reduce the likelihood of imbalances between study arms. Indoor host-seeking mosquitoes will be collected for 5 days each month using CDC UV light traps from 15 sentinel households. The CDC light trap will be placed next to the sleeping space of the study enrolled child and will be raised 1.5 m from the ground. The light trap will be set up at 7:00 p.m. and mosquitoes collected at 7:00 a.m. the next morning.

Indoor resting mosquitoes will be collected using pyrethrum spray catches (PSC) in the remaining 15 sentinel households in each arm of the study. The collections will be done inside the room where the child recruited into the study cohort sleeps. Mosquito sampling using PSC will be conducted early in the morning between 06:00 and 08:00 h. All the exit points to the sleeping area will be shut and a white sheet spread on the floor. An insecticide will then be sprayed in the room to knock down mosquitoes which will then be collected after 10 min. This sampling will be conducted 1 day for each month in the fixed sentinel households throughout the study period.

All the mosquito collections will be preserved in silica gel and transported to the respective national health laboratories where they will be stored in a freezer for identification using morphological and molecular methods. Morphological identification will use morphological keys for afro tropical vectors [48]. Molecular analysis of mosquito samples will be based on the polymerase chain reaction (PCR) to assess sporozoite infections in the mosquitoes and enzyme-linked immunosorbent assays (ELISA) for analysis of all blood-fed female *Anopheles* mosquitoes. A material transfer agreement (MTA) will be signed between the Ministries of Health of the project countries and *icipe* to allow for a sample of the collected mosquitoes to be transported to *icipe*, in Nairobi Kenya for quality assurance. In case there is no in-country capacity to process the collected samples, then all collected mosquitoes will be transported to *icipe* for morphological and molecular analysis.

##### *Cost-effectiveness endpoints*

To determine the cost-effectiveness of house screening, the costs associated with house screening will be collected from the implementing organizations (WHO and The NMCPs of the project countries). The per capita costs of house screening will be estimated by dividing the average cost of house screening per house by the household size, and this will be compared to the per capita costs of LLINs. Daily Adjusted Life Years (DALY) will be estimated for each household using standard methods on the basis of the number of malaria cases averted [49]. The cost-effectiveness ratio (CER) will be computed as the per capita costs of house screening per DALY averted.

The acceptability of house screening as a mosquito abatement tool will be evaluated using qualitative surveys that include key informant interviews and focus group discussions as the secondary endpoint.

#### Participant timeline {13}

#### Sample size {14}

##### *Clinical*

The sample size used in this study is based on simulation models described in *Hayes and Bennet* et al. for incidence rates [50]. Routine data collected from all health facilities in Nyimba district in Zambia estimated an incidence rate of 0.312 cases per person from January to June 2019. Using these estimates in Eq. 1 [50], we estimated that in order to detect a reduction of 35% on malaria incidence, with 80% power at the 5% significance level, 338 houses were required per study arm when following one child per household per year [51]. A total of 400 households with one child each were recruited per treatment arm. The additional households were enrolled to account for households lost to follow-up.

Data collected from all health facilities in Chokwe district, Mozambique, estimated an incidence rate of 0.325 cases per person from January to June. Using the same formula, we estimated that 324 children were required per treatment arm to observe a 35% treatment effect with 80% power at the 5% significance level [50]. A total of 400 households were also recruited in Mozambique.

1 $$ \mathrm{y}={\left({\mathrm{z}}_{\upalpha /2}+{\mathrm{z}}_{\upbeta}\right)}^2\left({\uplambda}_0+{\uplambda}_1\right)/{\left({\uplambda}_0\hbox{-} {\uplambda}_1\right)}^2 $$

##### *Entomological*

House screening is projected to reduce indoor malaria vector collections by at least 50% [51]. We used the *Hayes and* Bennet model for sample size calculation when using means to determine the number of households required to demonstrate a 50% reduction of indoor-entering mosquitoes associated with house screening, with 80% power at the 5% level of significance [50]. A study assessing different mosquito sampling schemes using light traps and human biting catches in Nyimba district, Zambia, in 2014, estimated that 2.6 *An. funestus* were caught using CDC light traps per night [52]. Using Eq. 2 below, we estimated that we would require 17 houses in each arm of the study. An additional 12 households were recruited for mosquito sampling to make a total of 30 households per study arm to allow for sampling of mosquitoes using both the CDC LTs and PSC techniques. Each sampling technique was used to sample mosquitoes from 15 households per study arm. As the standard deviation (SD) for this data was not reported, we assumed a SD of 1.96 for the sample size calculation.

Mosquito data from Chokwe in Mozambique was not available, and there we used the same sample size as that of Zambia of 30 households per treatment arm.

2 $$ \mathrm{n}={\left({\mathrm{z}}_{\upalpha /2}+{\mathrm{z}}_{\upbeta}\right)}^2\left({\sigma_0}^2+{\sigma_1}^2\right)/{\left({\upmu}_0\hbox{-} {\upmu}_1\right)}^2 $$

##### *Recruitment {15}*

Study participants will be recruited by the national project team in consultation with local health workers. The project staff will obtain verbal and written informed consent using ethical review committee (ERC)-approved language that outlines the objectives, benefits, and potential risks of the study and the data and biological samples that will be collected. Household heads will be briefed about the study, provided with an information sheet, and asked for their written consent to have their children involved in the study. Assent will be sought from all children above the age of 10 years. Household heads/guardians who do not want their children to participate in the study will be free to refuse participation. On the survey day, children will be informed about the survey procedures, making it clear that their participation is voluntary and that they might opt-out at any time if they chose to. Children unwilling to participate or whose parents did not give consent will be excluded from the selection procedure, with assent obtained from selected children before samples are collected. Participants’ names will be removed from the final database to ensure anonymity. Children with a positive malaria RDT will be treated for malaria according to national guidelines.

Participants will be free to withdraw from the study at any time without giving a reason. In the unlikely event that homeowners require their house to be returned to the pre-intervention state, items added will be removed. No replacements will be made during the surveillance period in either year, but in year 2, children who have withdrawn or are no longer in the study for any reason will be replaced by a child within the age range of 6 months—13 years in the same household. If another child within this age range is not present in that house, then another household with a child within this age range will be sampled in the same village. If a household withdraws consent, no further follow-up will be made in that household. All data collected up until the point of withdrawal will be kept for the per-protocol analysis at the end of the study. If the house was participating in the entomology collections, it will be replaced by a neighboring house of the same type and intervention status.

#### Assignment of interventions: allocation

##### *Sequence generation {16a}*

Households were used as the unit of randomization. For allocation of the households, a computer-generated list of random numbers was used. Households were randomly assigned following simple randomization procedures (computerized random numbers) to either the intervention or control group. The allocation was stratified by the village. The randomization sequence was created using Excel 2007 (Microsoft, Redmond, WA, USA) by the Co-PI’s in the project countries.

##### *Concealment mechanism {16b}*

All eligible households will be uploaded into an Ms Excel spreadsheet. The simple randomization formula will then be used to assign the households to the intervention and control groups. The Co-PI’s therefore did not know which household would be assigned to the intervention or control group before the allocation of the interventions.

##### *Implementation {16c}*

The simple randomization was done by a computer-generated random number list that was prepared by the Co-PI’s in each of the study countries. The randomization was stratified by the village so that there was an equal number of intervention and control households in each village.

#### Assignment of interventions: blinding

##### *Who will be blinded {17a}*

This study cannot be blinded since house screening will be visible to all including data collectors and the researchers involved. However, staff involved in reading the blood slides will be blinded to the intervention. Statisticians analyzing the primary endpoints of the study will also be blinded to the intervention.

##### *Procedure for unblinding if needed {17b}*

Datasets will be unblinded after all primary and secondary analysis have been conducted.

#### Data collection and management

##### *Plans for assessment and collection of outcomes {18a}*

Study endpoints will include clinical, entomological, and cost-effectiveness data. Data will be collected electronically using Open Data Kit (ODK) software which was programmed and uploaded on mobile phones. Once households have been enrolled in the study, a household survey adapted from a WHO standardized Malaria Indicator Survey (MIS) questionnaire (Supplementary material 1) will be administered to capture baseline demographic, behavioral, and socio-economic household indicators. Each household will be assigned a unique identification code at the time of questionnaire administration.

Baseline household data will be collected between April and October, after the long rains in southern Africa.

Entomological sampling will be undertaken throughout the study period and initiated after the baseline socio-demographic household data collection. Clinical malaria baseline data will also be collected after the household baseline survey (November-April) and in subsequent malaria transmission seasons at similar time points after the intervention implementation (Fig. 2).

##### *Plans to promote participant retention and complete follow-up {18b}*

Once a household and child are enrolled or randomized, the study team will make every reasonable effort to follow the child for the entire study period.

##### *Data management {19}*

Data will be stored by the NCMP/NMEC at their national offices in a secure database as they are the custodians of the *Demo* project. A unique ID number will be assigned to each participant. The NCMP/NMEC team will be responsible for overseeing data management. Data will be collected electronically and synced daily through 3G enabled Wi-Fi hotspot with the database at the NMCP/NMEC offices by the study supervisors. Collected data will remain on the tablets until the end of data collection to ensure that it is backed up. The NCMP/NMEC team will also ensure that the questionnaires uploaded to the database are backed up regularly to prevent any loss of data. At the end of the survey, all the data will be in an aggregated master database. The NCMP/NMEC team, WHO, and *icipe* staff will check the data for inconsistencies and will make necessary corrections.

After all, the primary and secondary analysis of the project data has been conducted, the database will be archived for the minimum NMCP/NMEC retention period and disposed of as per ERC guidelines. The ERC will be notified in writing of the intention to dispose of the project data by filling the ERC destruction form outlining the data disposal method and invited to witness the destruction of the hard disc that contains the project database.

##### *Confidentiality* {27}

Data confidentiality will be strictly kept with the data stored on an access-restricted/password-locked database accessible only to authorized personnel, and the data used for analysis will be de-identified so that they cannot be traced back to any individual. To maintain confidentiality, a system of coding will be used to link the name and data.

##### *Plans for collection, laboratory evaluation, and storage of biological specimens for genetic or molecular analysis in this trial/future use {33}*

During the cross-sectional surveys, clinical surveys will be done on all study children. A finger prick sample will be taken and an RDT used to test for the presence of malaria parasites. Thick and thin blood smears will be taken for later detection and quantification of *Plasmodium* parasites. Blood films will be stained in the field by project staff, transported to the in-country laboratory, and read independently by two microscopists blinded to the identity of the child. Any discrepancy will be resolved by a third senior microscopist. A sample of the blood films will be transported to *icipe* for quality assurance after signing the material transfer agreement (MTA) between *icipe* and the Ministries of Health of the Project countries. Children with malaria will be referred for treatment if they have not received any treatment already.

All the mosquito collections will be preserved in Eppendorf tubes with silica gel and transported to the laboratories where they will be identified to species level and examined for sporozoite infection and blood meal analysis. A sample of the mosquito specimen will also be transported to *icipe* for quality assurance. Blood slides and mosquito DNA will be stored in a −20°C non-frost-free freezer until DNA extraction. This data will be made available for future studies after all the guidelines, and conditions for secondary data use have been met at both *icipe* and the project countries.

### Statistical methods

#### Statistical methods for primary and secondary outcomes {20a}

A mixed-effects model will be used to compare clinical malaria incidence rates between the intervention and control arms, to allow for repeated measures within households, villages, and the effect of year. Possible confounders such as the age of child, gender, ethnicity, and season will be included in the models.

Differences in the abundance of mosquitoes caught indoors and outdoors will be made between the study aims to evaluate the impact of house screening on mosquito density. Mixed-effects models will be used to estimate differences in the numbers of mosquitoes, adjusting for repeated measures (i.e., within houses, villages), and other possible covariates such as the child’s age, gender, ethnicity, and rainfall. A Poisson distribution model with a log link function will be used, and a random factor for each data point will be included in the model to adjust for excess variation between data points (over-dispersion). All mean counts per treatment and their 95% confidence intervals (CIs) will be modeled as the exponential of the parameter estimates for models with no intercept included. No additional analysis will be conducted.

#### Interim analyses {21b}

Interim statistical data analyses will be implemented annually to monitor the progress of the project and a comprehensive data analysis will be implemented at the end of the project.

#### Methods in analysis to handle protocol non-adherence and any statistical methods to handle missing data {20c}

A per-protocol and an intention-to-treat analysis will be conducted.

#### Plans to give access to the full protocol, participant level-data, and statistical code {31c}

The study team members will have access to records. The authorized representatives of the sponsor, the ethics committee(s), or regulatory bodies may inspect all documents and records required to be maintained by the PI, including but not limited to medical records (office, clinic, or hospital) for the participants in this study. The clinical study site will permit access to such records. The results of the study will be made publicly available.

### Oversight and monitoring

#### Composition of the coordinating center and trial steering committee {5d}

A regional Project Steering Committee (PSC) of 6 members was established to provide oversight of this multi-country study. The PSC’s main mandate is the monitoring of the participating countries’ progress towards the implementation of the planned project activities as outlined in the protocol. The PSC will also ensure WHO and national patient safety guidelines are followed and adhered to, review reports on adverse events reported in the study, and patient’s rights are upheld.

#### Composition of the data monitoring committee, its role, and reporting structure {21a}

A DMSC will provide overall supervision of the study and ensure that it is conducted to the standards set out in Good Clinical Practice (https://www.mrc.ac.uk/documents/pdf/good-clinical-practice-in-clinical-trials/). In particular, the DSMC will concentrate on the progress of the trial, adherence to the protocol, patient safety, and the consideration of new information, and the SSC will formally report to the sponsor (UNEP). The DSMC will determine if additional interim analyses of trial data should be undertaken and assess any additional safety issues that may arise during the study. They will ensure the safety, rights, and well-being of the study participants and will report to the SCC at regular intervals.

#### Adverse event reporting and harms {22}

Adverse events and serious adverse events will be recorded during both transmission seasons. Sick children will receive referral notes to the nearest health facility, and treatment will be documented.

#### Frequency and plans for auditing trial conduct {23}

The trial implementation and progress will be reviewed yearly during the project steering committee meetings and way forward agreed upon.

#### Plans for communicating important protocol amendments to relevant parties (e.g., trial participants, ethical committees) {25}

In case of protocol amendments, this will be communicated to participants through community meetings and field assistants. This information will also be communicated to the DSMC through the established organogram.

#### Dissemination plans {31a}

The results of the study will be made publicly available through peer-reviewed publications and presentations at international conferences.

## Discussion

Progress made against malaria control has stalled in the past 5 years [53]. To get malaria control back on track, the WHO developed the Global Technical Strategy (GTS) for malaria 2016–2030 [4]. One of the core pillars of GTS is the universal coverage of at-risk populations with primary interventions [4]. However, to attain universal coverage with these core interventions, significant funding will be required [10]. It has recently been reported that the funding gap between the amount invested in malaria control to that required to stay on course has doubled since 2017 [53]. The global financial downturn recently witnessed will likely further widen this gap as donor organizations cut [54] or reprioritize funding meant for malaria control as a result of emerging public health threats such as the COVID-19 pandemic [5]. This turn of events indicates that this funding gap is unlikely to be met soon, and it is therefore unlikely that the GTS targets will be achieved. It is therefore prudent that countries that depend disproportionately on donor funding develop sustainable strategies for future malaria control.

In addition, recent evidence suggests that even with optimal coverage of core interventions, it will not be possible to eliminate malaria in high burden countries [55]. This is as a result of over-reliance on insecticide-based methods for malaria control which has led to the development of insecticide resistance and resilience to these interventions [55]. This occurrence further reinforces the argument for locally developed and sustainable malaria control if the GTS targets of malaria reduction are to be achieved.

House improvements have been shown to reduce malaria morbidity by half [22], and this study will evaluate whether house screening provides any additional protection against malaria compared to the sole use of LLINs. House screening presents a potential sustainable malaria control tool because, unlike current insecticide-based malaria interventions, house screening with non-insecticide-based materials can protect against insecticide-resistant vectors, a growing threat against malaria control [56]. Core malaria interventions predominantly rely on donor funding [53], and their coverage is consequently affected by fluctuations in the availability of these funds [10]. However, house screening using locally available materials and labor is unlikely to be affected by the availability of these funds and as such presents a sustainable solution to the challenges in resource-constrained settings.

Africa’s economy is experiencing rapid growth and with it is the exponential population growth and rapid urbanization [5]. With this development, the quality of housing is likely to improve, and this opportunity can be exploited to develop mosquito-proof houses at scale and thus impact malaria transmission downwards in these countries. House screening also presents a permanent intervention for mosquito control if implemented properly and with correct materials. As such, house screening is likely to present a cheaper malaria control intervention when compared to LLINs which must be replaced every few years. The one-time implementation of this intervention is likely to further improve the sustainability of house screening as a mosquito abatement tool.

This study will therefore assess the cost-effectiveness of the intervention while also exploring avenues through which the capital costs of house screening can be subsidized to promote its uptake. Further, a roadmap through which this intervention can be implemented will be explored and advocated by the community and project stakeholders.

This study, therefore, aims to provide the much-needed high-quality entomological and epidemiological evidence-based impact of house screening on malaria transmission from 3 well-designed randomized controlled trials from 3 eco-epidemiological settings in southern Africa. This study will provide high-quality data for evidence-based policy adoption of house screening a mosquito abatement tool.

### Trial status

This is protocol version 7. The participant recruitment started in February in Zambia 2019 and March 2019 in Mozambique. The recruitment process was completed in April and May 2019 for Zambia and Mozambique, respectively. This participant recruitment started in December 2020 in Zimbabwe and is currently ongoing. The delays in recruitment were due to the emergence of the COVID-19 pandemic.

## Supplementary Information

**Additional file 1.**

## Data Availability

Data confidentiality will be strictly kept with the data stored on an access-restricted/password-locked database accessible only to authorized personnel, and the data used for analysis will be de-identified so that they cannot be traced back to any individual.

## Abbreviations

ACT: Artemisinin combination therapy
DDT: Dichlorodiphenyltrichloroethane
DSMC: Data Management Steering Committee
ELISA: Enzyme-linked immunoabsorbent assay
GPS: Global Positioning System
GEF: Global Environment Fund
ICIPE: International Centre of Insect Physiology and Ecology
IEC: Information, education, communication
IRS: Indoor residual spraying
IVM: Integrated vector management
LLIN: Long-lasting insecticide-treated nets
NMCP: National Malaria Control Programme
NMEC: National Malaria Elimination Centre
NPC: National Project Coordinator
PCR: Polymerase chain reaction
RBM: Roll Back Malaria
RDT: Rapid diagnostic test
UNEP: United Nations Environment Programme
WHO: World Health Organization
WHO AFRO: World Health Organization African Region

## References

[1] WHO. World Malaria Report 2016 [Internet]. World Health Organization; 2016 [cited 2017 Oct 13]. Available from: http://apps.who.int/iris/bitstream/10665/252038/1/9789241511711-eng.pdf?ua=1

[2] Alonso P, Noor AM (2017). The global fight against malaria is at crossroads. Lancet. Elsevier.

[3] WHO. World malaria report 2019 [Internet]. 2019 [cited 2019 Dec 11]. p. 1–232. Available from: https://www.who.int/publications-detail/world-malaria-report-2019

[4] World Health Organization. Global technical strategy for malaria 2016–2030. Global Malaria Programme [Internet]. 2015 [cited 2021 Mar 12]. Available from: http://apps.who.int/iris/bitstream/handle/10665/176712/9789241564991_eng.pdf;jsessionid=66E6DA665C88369AF0BA3A99E8525283?sequence=1

[5] UNCTAD. Catalysing investment for transformative growth in africa.

[6] Cohen JM, Smith DL, Cotter C, Ward A, Yamey G, Sabot OJ, et al. Malaria resurgence: a systematic review and assessment of its causes. Malar J [Internet]. BioMed Central; 2012 [cited 2021 Mar 10];11:122. Available from: /pmc/articles/PMC3458906/10.1186/1475-2875-11-122 PMC3458906 22531245

[7] WHO Strategic Advisory Group on Malaria Eradication. Malaria eradication: benefits, future scenarios and feasibility. A report of the Strategic Advisory Group on Malaria Eradication [Internet]. Geneva World Heal. Organ. 2019. Available from: https://www.who.int/publications-detail/strategic-advisory-group-malaria-eradication-executive-summary

[8] Aborode AT, David KB, Uwishema O, Nathaniel AL, Imisioluwa JO, Onigbinde SB, et al. Fighting covid-19 at the expense of malaria in Africa: the consequences and policy options [Internet]. Am. J. Trop. Med. Hyg. American Society of Tropical Medicine and Hygiene; 2021 [cited 2021 Apr 9]. p. 26–9. Available from: https://www.who.int/malaria/

[9] World Health Organization. High burden to high impact: a targeted malaria response.

[10] Patouillard E, Griffin J, Bhatt S, Ghani A, Cibulskis R. Global investment targets for malaria control and elimination between 2016 and 2030 [Internet]. BMJ Glob. Heal. BMJ Publishing Group; 2017 [cited 2021 Mar 12]. p. 176. Available from: http://dx.doi.org/10. 10.1136/bmjgh-2016-000176 PMC5584487 29242750

[11] WHO. The Abuja declaration: ten years on [internet]. 2011. Available from: http://www.oecd.org/document/11/0,3746,en_2649_34447_44981579_1_1_1_1,00.html

[12] World Health Organization. Current health expenditure (CHE) as percentage of gross domestic product (GDP) (%) - data by country; Global Health Observatory repository. WHO. World Health Organization; 2020;

[13] RBM Partnership to End Malaria. RBM partnership strategic plan 2018–2020. 2018.

[14] Ogletree EJ. Megatrends: ten new directions transforming our lives John Naisbitt New York: Warner Books, 1982, 290 pp, $15.50. J Teach Educ [Internet]. SAGE Publications; 1983 [cited 2021 Mar 16];34:61–2. Available from: http://journals.sagepub.com/doi/10.1177/002248718303400516, 34, 5, 61, 62

[15] Barreaux P, Barreaux AMG, Sternberg ED, Suh E, Waite JL, Whitehead SA, et al. Priorities for broadening the malaria vector control tool kit [internet]. Trends Parasitol. Elsevier Ltd; 2017 [cited 2020 Oct 6]. p. 763–74. Available from: /pmc/articles/PMC5623623/?report=abstract 10.1016/j.pt.2017.06.003 PMC5623623 28668377

[16] Chanda E, Ameneshewa B, Bagayoko M, Govere JM, Macdonald MB. Harnessing integrated vector management for enhanced disease prevention [internet]. Trends Parasitol. Elsevier Ltd; 2017 [cited 2020 Oct 6]. p. 30–41. Available from: http://www.cell.com/article/S1471492216301623/fulltext 10.1016/j.pt.2016.09.006 27720141

[17] Huho B, Briët O, Seyoum A, Sikaala C, Bayoh N, Gimnig J, Okumu F, Diallo D, Abdulla S, Smith T, Killeen G Consistently high estimates for the proportion of human exposure to malaria vector populations occurring indoors in rural Africa. Int J Epidemiol [Internet]. 2013;42:235–247. Available from: https://academic.oup.com/ije/article/42/1/235/698545, DOI: 10.1093/ije/dys214 10.1093/ije/dys214 PMC3600624 23396849

[18] Bradley J, Lines J, Fuseini G, Schwabe C, Monti F, Slotman M, et al. Outdoor biting by Anopheles mosquitoes on Bioko Island does not currently impact on malaria control. Malar J [Internet]. BioMed Central Ltd.; 2015 [cited 2021 Jan 15];14:170. Available from: https://malariajournal.biomedcentral.com/articles/10.1186/s12936-015-0679-2 10.1186/s12936-015-0679-2 PMC4429929 25895674

[19] Burkina Faso southwest. Quantifying and characterizing hourly human exposure to malaria vectors bites in rural 1. [cited 2021 Jan 15]; Available from: 10.1101/2019.12.17.19014845 10.1186/s12889-021-10304-y PMC7847557 33516197

[20] Bayoh MN, Walker ED, Kosgei J, Ombok M, Olang GB, Githeko AK, et al. Persistently high estimates of late night, indoor exposure to malaria vectors despite high coverage of insecticide treated nets. Parasites and Vectors [Internet]. BioMed Central Ltd.; 2014 [cited 2021 Jan 15];7:380. Available from: http://parasitesandvectors.biomedcentral.com/articles/10.1186/1756-3305-7-380 10.1186/1756-3305-7-380 PMC4261540 25141761

[21] Killeen GF, Govella NJ, Mlacha YP, Chaki PP. Suppression of malaria vector densities and human infection prevalence associated with scale-up of mosquito-proofed housing in Dar es Salaam, Tanzania: re-analysis of an observational series of parasitological and entomological surveys. Lancet Planet Heal [Internet]. Elsevier; 2019 [cited 2019 Apr 1];3:e132–43. Available from: https://www.sciencedirect.com/science/article/pii/S254251961930035X?via%3Dihub 10.1016/S2542-5196(19)30035-X 30904112

[22] Tusting LS, Ippolito MM, Willey BA, Kleinschmidt I, Dorsey G, Gosling RD, et al. The evidence for improving housing to reduce malaria: a systematic review and meta-analysis. Malar J [Internet]. BioMed Central Ltd.; 2015 [cited 2020 Feb 12];14:209. Available from: https://malariajournal.biomedcentral.com/articles/10.1186/s12936-015-0724-1 10.1186/s12936-015-0724-1 PMC4460721 26055986

[23] Lindsay S, Emerson P, Parasitology JC-T in, 2002 undefined. Reducing malaria by mosquito-proofing houses [Internet]. Elsevier. 2002. Available from: http://parasites.trends.com 10.1016/s1471-4922(02)02382-6 12473368

[24] Manson P (2002). Classics of biology and medicine the mosquito-malaria THEORY-11 Experimental Proof of The Mosquito-Malaria Theorya.

[25] Kirby MJ, Ameh D, Bottomley C, Green C, Jawara M, Milligan PJ, Snell PC, Conway DJ, Lindsay SW Effect of two different house screening interventions on exposure to malaria vectors and on anaemia in children in The Gambia: a randomised controlled trial. Lancet. Elsevier; 2009;374:998–1009, 9694, DOI: 10.1016/S0140-6736(09)60871-0. 10.1016/S0140-6736(09)60871-0 PMC3776946 19732949

[26] Getawen SK, Ashine T, Massebo F, Woldeyes D, Lindtjørn B. Exploring the impact of house screening intervention on entomological indices and incidence of malaria in Arba Minch town, southwest Ethiopia: a randomized control trial. Acta Trop [Internet]. Elsevier B.V.; 2018 [cited 2021 Apr 22];181:84–94. Available from: https://pubmed.ncbi.nlm.nih.gov/29452110/10.1016/j.actatropica.2018.02.009 29452110

[27] Pinder M, Conteh L, Jeffries D, Jones C, Knudsen J, Kandeh B, et al. The RooPfs study to assess whether improved housing provides additional protection against clinical malaria over current best practice in The Gambia: study protocol for a randomized controlled study and ancillary studies. Trials [Internet]. BioMed Central Ltd.; 2016 [cited 2020 Feb 14];17:275. Available from: http://trialsjournal.biomedcentral.com/articles/10.1186/s13063-016-1400-7 10.1186/s13063-016-1400-7 PMC4891825 27255167

[28] Initiative PM. Zimbabwe - Malaria Operational Plan FY. 2018:2018.

[29] Sande S, Zimba M, Mberikunashe J, Tangwena A, Chimusoro A. Progress towards malaria elimination in Zimbabwe with special reference to the period 2003-2015. Malar J [Internet]. BioMed Central Ltd.; 2017 [cited 2020 Feb 21];16:295. Available from: http://malariajournal.biomedcentral.com/articles/10.1186/s12936-017-1939-0 10.1186/s12936-017-1939-0 PMC5525350 28738840

[30] Ministry of Health Zambia. ZAMBIA’S 2018 MALARIA INDICATOR SURVEY Perspective. 2017.

[31] Programme NME (2018). Republic of Zambia.

[32] Initiative M (2017). Zimbabwe Malaria Operational Plan FY 2017 [Internet].

[33] USAID Malaria Initiative. FY 2020 Mozambique Malaria Operational Plan [Internet]. Available from: www.pmi.gov

[34] Stevenson JC, Simubali L, Mbambara S, Musonda M, Mweetwa S, Mudenda T, et al. Detection of plasmodium falciparum infection in anopheles squamosus (diptera: Culicidae) in an area targeted for malaria elimination, Southern Zambia. J Med Entomol. Entomological Society of America; 2016;53:1482–1487. 10.1093/jme/tjw091 PMC5106822 27297214

[35] PMI | Africa IRS (AIRS) Project. Entomological activities annual report [internet]. 2016. Available from: www.abtassociates.com

[36] Lobo NF, St. Laurent B, Sikaala CH, Hamainza B, Chanda J, Chinula D, et al. Unexpected diversity of Anopheles species in Eastern Zambia: implications for evaluating vector behavior and interventions using molecular tools. Sci Rep. Nature Publishing Group; 2015;5, 1, DOI: 10.1038/srep17952. 10.1038/srep17952 PMC4673690 26648001

[37] Fornadel CM, Norris LC, Franco V, Norris DE. Unexpected anthropophily in the potential secondary malaria vectors anopheles coustani s.l. and anopheles squamosus in Macha, Zambia. Vector-Borne Zoonotic Dis. Mary Ann Liebert, Inc.; 2011;11:1173–9. 10.1089/vbz.2010.0082 PMC3151625 21142969

[38] Wagman JM, Varela K, Zulliger R, Saifodine A, Muthoni R, Magesa S, et al. Reduced exposure to malaria vectors following indoor residual spraying of pirimiphos-methyl in a high-burden district of rural Mozambique with high ownership of long-lasting insecticidal nets: entomological surveillance results from a cluster-randomized trial. Malar J [Internet]. BioMed Central Ltd; 2021 [cited 2021 Mar 31];20:54. Available from: https://malariajournal.biomedcentral.com/articles/10.1186/s12936-021-03583-8 10.1186/s12936-021-03583-8 PMC7819201 33478533

[39] Chanda E, Hemingway J, Kleinschmidt I, Rehman AM, Ramdeen V, Phiri FN, Coetzer S, Mthembu D, Shinondo CJ, Chizema-Kawesha E, Kamuliwo M, Mukonka V, Baboo KS, Coleman M Insecticide resistance and the future of malaria control in Zambia. Gosling RD, editor. PLoS One [Internet]. Public Library of Science; 2011 [cited 2020 Oct 6];6:e24336. Available from: https://dx.plos.org/10.1371/journal.pone.0024336, 6, 9, e24336 10.1371/journal.pone.0024336 PMC3167838 21915314

[40] Redditt V, MOle---- Moiyoi K, Rodriguez W, Rosenberg J, Weintraub R. Cases in global health delivery [internet]. Available from: www.globalhealthdelivery.org

[41] Zambia CSO. Census of population and housing preliminary report [Internet]. 2010:2011 Available from: http://unstats.un.org/unsd/demographic/sources/census/wphc/Zambia/PreliminaryReport.pdf.

[42] Hamainza B, Sikaala CH, Moonga HB, Chanda J, Chinula D, Mwenda M, et al. Incremental impact upon malaria transmission of supplementing pyrethroid-impregnated long-lasting insecticidal nets with indoor residual spraying using pyrethroids or the organophosphate, pirimiphos methyl. Malar J [Internet]. BioMed Central Ltd.; 2016 [cited 2021 Mar 17];15:100. Available from: http://www.malariajournal.com/content/15/1/100 10.1186/s12936-016-1143-7 PMC4758014 26893012

[43] World Weather Online. Nyimba, North-Western, Zambia weather averages|monthly average high and low temperature|average precipitation and rainfall days|world weather online [internet]. [cited 2021 Mar 31]. Available from: https://www.worldweatheronline.com/nyimba-weather-averages/north-western/zm.aspx

[44] Zengenene MP, Soko W, Brooke BD, Koekemoer LL, Govere J, Mazarire TT (2020). Anopheles species composition and breeding habitat characterisation in Chiredzi District. Zimbabwe. African entomol. Entomological Society of Southern Africa.

[45] Cuamba N, Morgan JC, Irving H, Steven A, Wondji CS. High level of pyrethroid resistance in an Anopheles funestus population of the Chokwe District in Mozambique. Gilbert MTP, editor. PLoS One [Internet]. Public Library of Science; 2010 [cited 2021 Apr 1];5:e11010. Available from: https://dx.plos.org/10.1371/journal.pone.0011010, 5, 6, e11010 10.1371/journal.pone.0011010 PMC2882342 20544036

[46] Ministry of Health Zambia. Guidelines for diagnosis and treatment of malaria in Zambia. [Internet]. Lusaka; 2017. Available from: https://static1.squarespace.com/static/58d002f017bffcf99fe21889/t/5cb9738a42ffca0001aaedbc/1555657626933/NationalMalariaTreatmentGuidelines2017_Final20170917+%281%29.pdf

[47] WHO. For the treatment of malaria guidelines [internet]. WHO; 2015 [cited 2020 Sep 7]. Available from: www.who.int

[48] Coetzee M. Key to the females of Afrotropical Anopheles mosquitoes (Diptera: Culicidae). Malar J. BioMed Central Ltd.; 2020;19. 10.1186/s12936-020-3144-9 PMC7020601 32054502

[49] Gunda R, Chimbari MJ. Cost-effectiveness analysis of malaria interventions using disability adjusted life years: a systematic review [Internet]. Cost Eff. Resour. Alloc. BioMed Central Ltd.; 2017 [cited 2021 Apr 6]. Available from: https://pubmed.ncbi.nlm.nih.gov/28680367/10.1186/s12962-017-0072-9 PMC5494144 28680367

[50] Hayes RJ, Bennett S. Simple sample size calculation for cluster-randomized trials. Int. J. Epidemiol. 1999, 28, 2, 319, 326, DOI: 10.1093/ije/28.2.319. 10.1093/ije/28.2.319 10342698

[51] Pinder M, Conteh L, Jeffries D, Jones C, Knudsen J, Kandeh B, et al. The RooPfs study to assess whether improved housing provides additional protection against clinical malaria over current best practice in The Gambia: study protocol for a randomized controlled study and ancillary studies. Trials [Internet]. BioMed Central Ltd.; 2016 [cited 2020 Mar 3];17:275. Available from: http://trialsjournal.biomedcentral.com/articles/10.1186/s13063-016-1400-7 10.1186/s13063-016-1400-7 PMC4891825 27255167

[52] Sikaala CH, Chinula D, Chanda J, Hamainza B, Mwenda M, Mukali I, et al. A cost-effective, community-based, mosquito-trapping scheme that captures spatial and temporal heterogeneities of malaria transmission in rural Zambia. Malar J. BioMed Central Ltd.; 2014;13. 10.1186/1475-2875-13-225 PMC4060139 24906704

[53] World Health Organization. World malaria report 2020: 20 years of global progress and challenges [Internet]. 2020 [cited 2021 Apr 8]. Available from: https://www.wipo.int/amc/en/

[54] United Kingdom Research and Innovation. UKRI Official Development Assistance letter 11 March 2021 – UKRI [Internet]. [cited 2021 Apr 8]. Available from: https://www.ukri.org/our-work/ukri-oda-letter-11-march-2021/

[55] Killeen GF. Characterizing, controlling and eliminating residual malaria transmission. Malar J [Internet]. BioMed Central; 2014 [cited 2019 Mar 28];13:330. Available from: https://malariajournal.biomedcentral.com/articles/10.1186/1475-2875-13-330 10.1186/1475-2875-13-330 PMC4159526 25149656

[56] Ranson H, Raphael N, Lines J, Moiroux N, Nkuni Z, Corbel V. Pyrethroid resistance in African anopheline mosquitoes: what are the implications for malaria control? Trends Parasitol [Internet]. Elsevier Current Trends; 2011 [cited 2017 Oct 13];27:91–8. Available from: http://www.sciencedirect.com/science/article/pii/S1471492210001753 10.1016/j.pt.2010.08.004 20843745

