# Supplementary material for: Evaluating the efficacy, impact, and feasibility of community-based house screening as a complementary malaria control intervention in southern Africa: a study protocol for a household randomized trial
Source: Trials. 2021 Dec 6;22:883. doi: 10.1186/s13063-021-05768-7 (PMC8646012; doi:10.1186/s13063-021-05768-7)
Supplement: Supplementary file 1 — Additional file 1. [file 13063_2021_5768_MOESM1_ESM.pdf]

1.1 Household questionnaire

IDENTIFICATION[1]	
PLACE NAME ______________________	
NAME OF HOUSEHOLD HEAD ______________________	
CLUSTER NUMBER..	___ ___ ___
HOUSEHOLD NUMBER..	___ ___
REGION..	___ ___
URBAN/RURAL (URBAN=1, RURAL=2)..	___
LARGE CITY/SMALL CITY/TOWN/COUNTRYSIDE[2].. (LARGE CITY=1, SMALL CITY=2, TOWN=3, COUNTRYSIDE=4)	___

INTERVIEWER VISITS				
	1	2	3	FINAL VISIT
DATE	________	________	________	DAY ___ ___<br>MONTH ___ ___<br>YEAR ___ ___ ___ ___
INTERVIEWER'S NAME	________	________	________	NAME ___ ___
RESULT*	________	________	________	RESULT ___

INTERVIEWER VISITS				
NEXT VISIT: DATE<br>TIME				TOTAL NO. OF VISITS
*RESULT CODES:<br>1 COMPLETED<br>2 NO HOUSEHOLD MEMBER AT HOME OR NO COMPETENT RESPONDENT AT HOME AT TIME OF VISIT<br>3 ENTIRE HOUSEHOLD ABSENT FOR EXTENDED PERIOD OF TIME<br>4 POSTPONED<br>5 REFUSED<br>6 DWELLING VACANT OR ADDRESS NOT A DWELLING<br>7 DWELLING DESTROYED<br>8 DWELLING NOT FOUND<br>9 OTHER ______<br>(SPECIFY)				TOTAL PERSONS IN HOUSEHOLD<br>TOTAL ELIGIBLE WOMEN<br>LINE NUMBER OF RESPONDENT TO HOUSEHOLD QUESTIONNAIRE

SUPERVISOR	OFFICE EDITOR	KEYED BY
NAME ______<br>DATE ______		

[1] This section should be adapted for country-specific survey design.

[2] The following guidelines should be used to categorize urban sample points: “Large cities” are national capitals and places with over 1 million population; “small cities” are places with between 50,000 and 1 million population; the remaining urban sample points are “towns.”

HOUSEHOLD LISTING

Now we would like some information about the people who usually live in your household or who are staying with you now.

LINE NO.	USUAL RESIDENTS AND VISITORS	RELATIONSHIP TO HEAD OF HOUSEHOLD	SEX	EDUCATION		RESIDENCE		AGE	TIME INDOORS / OUTDOORS				ELIGIBLE WOM
	Please give me the names of the persons who usually live in your household and guests of the household who stayed here last night, starting with the head of the household.	What is the relationship of (NAME) to the head of the household? *	Is (NAME) male or female?	Is (NAME). attending formal school? 1 = Yes 0 = No	What is the highest level of formal education (NAME) completed? (see **code 2** below)	Does (NAME) usually live here?	Did (NAME) stay here last night?	How old is (NAME)?	To the nearest hour, what time last night did (NAME) go indoors for the evening? RECORD HOUR ON 24 HOUR CLOCK	To the nearest hour, what time last night did (NAME) go to bed? RECORD HOUR ON 24 HOUR CLOCK	To the nearest hour, what time this morning did (NAME) get out of bed? RECORD HOUR ON 24 HOUR CLOCK	To the nearest hour, what time this morning did (NAME) go outdoors? RECORD HOUR ON 24 HOUR CLOCK	CIRCLE LINE NUMBER OF AL WOMEN AGE 1
(1)	(2)	(3)	(4)	(5.1)	(5.2)	(6)	(7)	(8)	(9.1)	(9.2)	(9.3)	(9.4)	(10)
			M F			YES NO	YES NO	IN YEARS	TIME (24 Hours)	TIME (24 Hours)	TIME (24 Hours)	TIME (24 Hours)	
01			1 2	YES NO 1 2		1 2	1 2						01
02			1 2	YES NO 1 2		1 2	1 2						02
03			1 2	YES NO		1 2	1 2						03

				1 2									
04			1 2	YES NO<br>1 2		1 2	1 2						04
05			1 2	YES NO<br>1 2		1 2	1 2						05

* CODES FOR Q.3

RELATIONSHIP TO HEAD OF HOUSEHOLD:

01 = HEAD

02 = WIFE/HUSBAND

03 = SON OR DAUGHTER

04 = SON-IN-LAW OR DAUGHTER-IN-LAW

05 = GRANDCHILD

06 = PARENT

07 = PARENT-IN-LAW

08 = BROTHER OR SISTER

09 = OTHER RELATIVE

10 = ADOPTED/FOSTER/ STEPCHILD

11 = NOT RELATED

98 = DON'T KNOW

* CODES FOR Q.5.2

0= None
1 =Sub-standard A or B; Grade 1
2 =Standard 1: Grade 2
3 =Standard 2; Grade 3
4 =Standard 3; Grade 4
5 =Standard 4; Grade 5
6 =Standard 5; Grade 6
7 =Standard 6; Grade 7
8 =Form 1; Grade 8
9 =Form 2; Grade 9
10 =Form 3; Grade 10
11 =Form 4; Grade 11
12 =Form 5; Grade 12
13 =Form 6 Lower
14 =Form 6 Upper
15= College Student
16= Undergraduate student
17= Certificate/Diploma
18 =Bachelors Degree
19 =Masters Degree & Above

LINE NO.	FEVER PREVALENCE AND TREATMENT									
	Has (NAME) been ill with a fever at any time in the last 2 weeks?<br><br>IF NO SKIP TO NEXT PERSON,	How many days ago did the fever start?<br><br>IF LESS THAN ONE DAY, THEN RECORD '00'.	Did (NAME) seek advice or treatment for the fever from any source?	Where did you seek advice or treatment?<br><br>Anywhere else?<br><br>RECORD ALL SOURCES MENTIONED	How many days after the fever began did (NAME) first seek advice or treatment?<br><br>IF SAME DAY, RECORD '00'.	Is (NAME) still sick with a fever?	At any time during the illness, did (NAME) take any drugs for the fever?	What drugs did (NAME) take?[1]<br><br>Any other drugs?<br><br>RECORD ALL MENTIONED.	How long after the fever started did (NAME) first take DRUG NAME?	For how many days did (NAME) take the DRUG NAME?<br><br>IF 7 OR MORE DAYS, RECORD '7'
(1)	(9.1)	(9.2)	(9.3)	(9.4)	(9.5)	(9.6)	(9.7)	(9.8)	(9.9)	(9.10)
01	YES NO DK<br><br>1 2 8	DK = 99<br><br>|░|░|	YES NO DK<br><br>1 2 8	PUBLIC SECTOR<br>GOVT. HOSPITAL…………….A<br>GOVT. HEALTH CENTER…….B<br>GOVT. HEALTH POST ……..C<br>MOBILE CLINIC………………D<br>FIELD WORKER………………F<br>OTHER PUBLIC ____________G (SPEC)<br>PRIVATE MEDICAL SECTOR<br>PVT. HOSPITAL/CLINIC……………..H<br>PHARMACY ………………………..…I<br>PRIVATE DOCTOR……………………J	DK = 99<br><br>DAYS…..<br><br>|░|░|	YES NO DK<br><br>1 2 8	YES NO DK<br><br>1 2 8	DK = 8<br><br>SP/FANSIDAR……A<br>QUININE…………..B<br>COARTEM………..C<br>DHAP………………D<br>ASPIRIN……………E<br>PARACETAMOL....F<br>IBUPROFEN……...G<br>OTHER _______....X<br>DON'T KNOW…….Z	DK = 8<br><br>SAME DAY…………………….0<br>NEXT DAY…………………….1<br>2 DAYS AFTER THE FEVER..2<br>3 DAYS AFTER THE FEVER..3<br>4 OR MORE DAYS<br>AFTER THE FEVER……..4<br>DON'T KNOW ….8	DK = 99<br><br>|░|░|

				MOBILE CLINIC……………………….K<br>FIELD WORKER……………………….L<br>OTHER PVT. MED. ________M (SPECIY)<br>OTHER SOURCE<br>SHOP..N<br>TRAD. PRACTITIONER..............O<br>OTHER ______________ X (SPECIFY)						
02	1 2 8		1 2 8	PUBLIC SECTOR<br>GOVT. HOSPITAL…………….A<br>GOVT. HEALTH CENTER…….B<br>GOVT. HEALTH POST ……..C<br>MOBILE CLINIC………………D<br>FIELD WORKER………………F<br>OTHER PUBLIC ____________G (SPEC)<br>PRIVATE MEDICAL SECTOR<br>PVT. HOSPITAL/CLINIC…………….H<br>PHARMACY ………………………..…I<br>PRIVATE DOCTOR……………………J<br>MOBILE CLINIC……………………….K<br>FIELD WORKER……………………….L<br>OTHER PVT. MED. ________M (SPECIY)<br>OTHER SOURCE<br>SHOP..N<br>TRAD. PRACTITIONER..............O<br>OTHER ______________ X (SPECIFY)	DAYS…..	1 2 8	1 2 8	SP/FANSIDAR……A<br>QUININE…………..B<br>COARTEM………..C<br>DHAP………………D<br>ASPIRIN……………E<br>PARACETAMOL....F<br>IBUPROFEN……..G<br>OTHER _______....X<br>DON'T KNOW…….Z	SAME DAY…………………….0<br>NEXT DAY…………………….1<br>2 DAYS AFTER THE FEVER..2<br>3 DAYS AFTER THE FEVER..3<br>4 OR MORE DAYS<br>AFTER THE FEVER……..4<br>DON'T KNOW ….8	

03	1 2 8	|░|░|	1 2 8	PUBLIC SECTOR<br>GOVT. HOSPITAL……………..A<br>GOVT. HEALTH CENTER…….B<br>GOVT. HEALTH POST ……..C<br>MOBILE CLINIC………………D<br>FIELD WORKER………………F<br>OTHER PUBLIC ___________G (SPEC)<br>PRIVATE MEDICAL SECTOR<br>PVT. HOSPITAL/CLINIC……………..H<br>PHARMACY …………………………..I<br>PRIVATE DOCTOR……………………J<br>MOBILE CLINIC………………………K<br>FIELD WORKER………………………L<br>OTHER PVT. MED. ________M (SPECIY)<br>OTHER SOURCE<br>SHOP..N<br>TRAD. PRACTITIONER..............O<br>OTHER _____________ X (SPECIFY)	DAYS…..<br>|░|░|	1 2 8	1 2 8	SP/FANSIDAR……A<br>QUININE…………..B<br>COARTEM………...C<br>DHAP………………D<br>ASPIRIN……………E<br>PARACETAMOL....F<br>IBUPROFEN……..G<br>OTHER _______....X<br>DON'T KNOW…….Z	SAME DAY……………………0<br>NEXT DAY……………………1<br>2 DAYS AFTER THE FEVER..2<br>3 DAYS AFTER THE FEVER..3<br>4 OR MORE DAYS<br>AFTER THE FEVER……..4<br>DON'T KNOW ….8	|░|░|
04	1 2 8	|░|░|	1 2 8	PUBLIC SECTOR<br>GOVT. HOSPITAL……………..A<br>GOVT. HEALTH CENTER…….B<br>GOVT. HEALTH POST ……..C<br>MOBILE CLINIC………………D<br>FIELD WORKER………………F<br>OTHER PUBLIC ___________G (SPEC)	DAYS…..<br>|░|░|	1 2 8	1 2 8	SP/FANSIDAR……A<br>QUININE…………..B<br>COARTEM………...C<br>DHAP………………D<br>ASPIRIN……………E<br>PARACETAMOL....F<br>IBUPROFEN……..G	SAME DAY……………………0<br>NEXT DAY……………………1<br>2 DAYS AFTER THE FEVER..2<br>3 DAYS AFTER THE FEVER..3<br>4 OR MORE DAYS<br>AFTER THE FEVER……..4<br>DON'T KNOW ….8	|░|░|

				PRIVATE MEDICAL SECTOR<br>PVT. HOSPITAL/CLINIC……………..H<br>PHARMACY ………………………..…I<br>PRIVATE DOCTOR……………………J<br>MOBILE CLINIC………………………K<br>FIELD WORKER………………………L<br>OTHER PVT. MED. ________M (SPECIY)<br>OTHER SOURCE<br>SHOP..N<br>TRAD. PRACTITIONER..............O<br>OTHER ______________ X (SPECIFY)				OTHER _______....X<br>DON'T KNOW…….Z		
05	1 2 8	|░|░|	1 2 8	PUBLIC SECTOR<br>GOVT. HOSPITAL…………….A<br>GOVT. HEALTH CENTER…….B<br>GOVT. HEALTH POST ……..C<br>MOBILE CLINIC………………D<br>FIELD WORKER………………F<br>OTHER PUBLIC ___________G (SPEC)<br>PRIVATE MEDICAL SECTOR<br>PVT. HOSPITAL/CLINIC……………..H<br>PHARMACY ………………………..…I<br>PRIVATE DOCTOR……………………J<br>MOBILE CLINIC………………………K<br>FIELD WORKER………………………L<br>OTHER PVT. MED. ________M (SPECIY)	DAYS….. |░|░|	1 2 8	1 2 8	SP/FANSIDAR……A<br>QUININE…………..B<br>COARTEM………..C<br>DHAP………………D<br>ASPIRIN……………E<br>PARACETAMOL....F<br>IBUPROFEN……..G<br>OTHER _______....X<br>DON'T KNOW…….Z	SAME DAY……………………0<br>NEXT DAY……………………1<br>2 DAYS AFTER THE FEVER..2<br>3 DAYS AFTER THE FEVER..3<br>4 OR MORE DAYS<br>AFTER THE FEVER……..4<br>DON'T KNOW ….8	|░|░|

				OTHER SOURCE<br>SHOP..N<br>TRAD. PRACTITIONER..............O<br>OTHER _______________ X (SPECIFY)						

TICK HERE IF CONTINUATION SHEET USED [ ]

Just to make sure that I have a complete listing:

1)	Are there any other persons such as small children or infants that we have not listed?	YES	[ ] ->	ENTER EACH IN TABLE	NO	[ ]
2)	In addition, are there any other people who may not be members of your family, such as domestic staff, lodgers or friends who usually live here?	YES	[ ] ->	ENTER EACH IN TABLE	NO	[ ]
3)	Are there any guests or temporary visitors staying here, or anyone else who stayed here last night, who have not been listed?	YES	[ ] ->	ENTER EACH IN TABLE	NO	[ ]

NO.	QUESTIONS AND FILTERS	CODING CATEGORIES	SKIP
9.11	For the head of household, did he/she ever **attend** school?	YES .... 1<br>NO 2	 →10
9.12	For the head of household, what is the highest level of school **attended**: primary, secondary, or higher?[1]	PRIMARY .... 1<br>SECONDARY .... 2<br>HIGHER 3	
10	What is the main source of drinking water for members of your household?[1]	PIPED WATER<br>PIPED INTO DWELLING .... 11<br>PIPED INTO YARD/PLOT .... 12<br>PUBLIC TAP/STANDPIPE .... 13<br>TUBE WELL OR BOREHOLE .... 21<br>DUG WELL<br>PROTECTED WELL .... 31<br>UNPROTECTED WELL .... 32<br>WATER FROM SPRING<br>PROTECTED SPRING .... 41<br>UNPROTECTED SPRING .... 42<br>RAINWATER 51	DISTANCE TO WATER SOURCE (MINS)

		TANKER TRUCK .... 61<br>CART WITH SMALL TANK .... 71<br>SURFACE WATER (RIVER/DAM/<br>LAKE/POND/STREAM/CANAL/<br>IRRIGATION CHANNEL .... 81<br>BOTTLED WATER................................ 91<br><br>OTHER ______________________ 96<br>(SPECIFY)	

11	What kind of toilet facility does your household use?[1]	FLUSH OR POUR FLUSH TOILET<br>FLUSH TO PIPED SEWER<br>SYSTEM ....11<br>FLUSH TO SEPTIC TANK..................12<br>FLUSH TO PIT LATRINE ....13<br>FLUSH TO SOMEWHERE ELSE ....14<br>FLUSH, DON'T KNOW WHERE.........15<br>PIT LATRINE<br>VENTILATED IMPROVED<br>PIT LATRINE (VIP).........................21<br>PIT LATRINE WITH SLAB..................22<br>PIT LATRINE WITHOUT SLAB/<br>OPEN PIT.......................................23<br>COMPOSTING TOILET..........................31<br>BUCKET TOILET ....41<br>HANGING TOILET/HANGING<br>LATRINE ....51<br>NO FACILITY/BUSH/FIELD....................61<br><br>OTHER ___________________________ 96<br>(SPECIFY)	

12a	Does your household have:[2]		
		YES NO	
	Electricity?	ELECTRICITY 1 2	
	A radio?	RADIO.. 1 2	
	A television?	TELEVISION 1 2	
	A mobile telephone?	MOBILE TELEPHONE 1 2	
	A non-mobile telephone?	NON-MOBILE TELEPHONE…….1 2	
	A refrigerator?	REFRIGERATOR........................ 1 2	
	A bed?	BED.. 1 2	
	A chair?	CHAIR……………………….…..1 2	
	A table?	TABLE.. 1 2	
	A Cupboard?	CUPBOARD................................ 1 2	
	A sofa?	SOFA.……………………….…..1 2	
	A clock?	CLOCK....................................... 1 2	
	A fan?	FAN.. 1 2	
	A sewing machine?	SEWING MACHINE.…………..….1 2	
	A cassette player?	CASSETTE PLAYER 1 2	
	A plough?	PLOUGH.……….……………..….1 2	
	A grain grinder?	GRAIN GRINDER........................ 1 2	
	A VCR/DVD?	VCR/DVD 1 2	
	A tractor?	TRACTOR.………….……….…..1 2	
	A vehicle?	VEHICLE.................................... 1 2	
	A hammer mill?	HAMMER MILL 1 2	

13	What type of fuel does your household mainly use for cooking?	ELECTRICITY ....01<br>LPG/NATURAL GAS ....02<br>BIOGAS ....03<br>KEROSENE ....04<br>COAL/LIGNITE.......................................05<br>CHARCOAL ....06<br>FIREWOOD/STRAW..............................07<br>DUNG ....08<br><br>OTHER ___________________________ 96<br>(SPECIFY)	

[1] Coding categories to be developed locally and revised based on the pretest; however, the broad categories must be maintained.

[2] Additional indicators of socioeconomic status should be added, especially to distinguish among lower socioeconomic classes.

NO.	QUESTIONS AND FILTERS	CODING CATEGORIES	SKIP
14a	MAIN MATERIAL OF THE FLOOR.[1]<br><br>RECORD OBSERVATION.	NATURAL FLOOR<br>EARTH/SAND ....11<br>DUNG...12<br>RUDIMENTARY FLOOR<br>WOOD PLANKS.................................21<br>PALM/BAMBOO.................................22<br>FINISHED FLOOR<br>PARQUET OR POLISHED WOOD.....31<br>VINYL OR ASPHALT STRIPS............32<br>CERAMIC TILES................................33<br>CEMENT..34<br>CARPET...35<br><br>OTHER ________________________ 96<br>(SPECIFY)	

14b	MAIN MATERIAL OF THE WALL.[1]<br><br>RECORD OBSERVATION.	NATURAL WALL<br>No walls....11<br>Cane/sticks/bamboo/reed....12<br>RUDIMENTARY WALL<br>Bamboo/wood with mud....21<br>Stone with mud....22<br>Uncovered abode....23<br>Plywood....24<br>Carton....25<br>FINISHED WALL<br>Cement....31<br>Stone with lime/cement....32<br>Bricks....33<br>Cement blocks....34<br>Covered Abode....35<br>Wood planks/shingles....36<br><br>OTHER ____________ 96<br>(SPECIFY)	

14c	MAIN MATERIAL OF THE ROOF.[1]<br><br>RECORD OBSERVATION.	NATURAL ROOF<br>Thatch/Leaf ....11<br>Sticks and mud...................................12<br>RUDIMENTARY ROOF<br>Rustic mat/plastic sheet......................21<br>Reed/bamboo.....................................22<br>Wood planks.......................................23<br>FINISHED WALL<br>Corrugated iron ....31<br>Wood..32<br>Calamine/cement fibre........................33<br>Cement/concrete ....34<br>Roofing shingles.................................35<br><br>OTHER ________________________ 96<br>(SPECIFY)	
14c1	ARE THE EAVES OF THE HOUSE OR BUILDING OCCUPIED BY THIS HOUSEHOLD OPEN OR CLOSED?<br><br>RECORD OBSERVATION.	OPEN………………………… 1<br>CLOSED ………………………2<br>PARTIALLY OPEN……………3	
14c2	DOES THE PART OF THE HOUSE OR BUILDING OCCUPIED BY THE HOUSEHOLD HAVE A CEILING?<br><br>RECORD OBSERVATION.	NONE………………………… 1<br>PARTIAL/POORLY SEALED/WORN OUT……………………2<br>COMPLETE AND SEALED…………3	

14c3	IF A CEILING IS PRESENT, WHAT TYPE OF MATERIAL IS THE CEILING PRIMARILY CONSTRUCTED OF?<br><br>RECORD OBSERVATION.	WOOD / PLYWOOD BOARDS…………………………. 1<br>GYPSUM / PLASTERCASTE………………………..2<br>MUD AND WADDLE……..……………..3<br>WOVEN PALM THATCH……..…………4<br>OTHER……...……………..5	
14d	TYPE OF WINDOWS<br><br>RECORD OBSERVATION.	YES NO<br>ANY WINDOW…………………1 2<br>WINDOWS WITH GLASS....... 1 2<br>WINDOWS WITH SCREENS...1 2<br>WINDOWS WITH CURTAINS OR SHUTTERS…………....1 2	
14d1	Are the windows and any airbrick gaps in the house or building boarded up, glazed or screened against mosquito entry with netting ?<br><br>ASK OR RECORD OBSERVATION.	COMPLETE…………….……………. 1<br>COMPLETE WITH HOLES…………………………….…...…2<br>INCOMPLETE OR BADLY DAMAGED…3<br>ABSENT……………………………………4	—<14e
14d2	If windows are boarded up, glazed or screened, what primary material is used to do so ?<br><br>ASK OR RECORD OBSERVATION.	WOOD BOARDS……………………. 1<br>GLASS……………………………….……..2<br>METAL NETTING…..……..……………..3<br>FABRIC NETTING……..……..…………4<br>PLASTIC NETTING……..……...…………5<br>OTHER……...……………..6	—<14e

14e	How many separate rooms are in this household?<br><br>INCLUDE ALL ROOMS, INCLUDING KITCHEN, TOILET, SLEEPING ROOMS, SALON, etc.	NUMBER OF ROOMS ☐☐<br>L	

14f	How many rooms in this household are used for sleeping?<br><br>INCLUDE ONLY ROOMS WHICH ARE USUALLY USED FOR SLEEPING.	NUMBER OF SLEEPING ROOMS	
14g	How many separate sleeping spaces are there in your household?<br><br>INCLUDE ALL SLEEPING SPACES, INCLUDING IF THERE IS MORE THAN ONE SLEEPING SPACE IN EACH ROOM USED FOR SLEEPING.	NUMBER OF SLEEPING SPACES	
14h	Does any member of the household own any agricultural land?	YES...1<br>NO...2	—<14j
14i	How much agricultural land do members of this household own?	Lima……………………..<br>Acres……………………..<br>Hectares…………………<br><br>95 or more hectares…………….995<br>Don't know………………………998	
14j	Does this household own any livestock, herds other farm animals, or poultry?	YES...1<br>NO...2	

14k	How many of the following animals does this household own?<br>IF NONE, ENTER '0'<br>IF MORE THAN 95, ENTER '95'<br>IF UNKNOWN, ENTER '98':<br>Traditional cattle?<br>Dairy cattle?<br>Beef cattle?<br>Horses, donkeys, mules?<br>Goats?<br>Sheep?<br>Pigs?<br>Chickens?<br>Other poultry?<br>Other livestock?	TRADITIONAL |__|__| DAIRY |__|__| BEEF |__|__| HORSES/DONKEYS/MULES |__|__| GOATS |__|__| SHEEP |__|__| PIGS |__|__| CHICKEN |__|__| OTHER POULTRY |__|__| OTHER LIVESTOCK |__|__|	

15	Does any member of your household own:<br><br>A watch?<br>A bicycle?<br>A motorcycle or motor scooter?<br>An animal drawn cart?<br>A car or truck?<br>A boat with a motor?<br>A banana boat?	YES NO<br>WATCH..1 2<br>BICYCLE......................................1 2<br>MOTORCYCLE/SCOOTER.........1 2<br>ANIMAL-DRAWN CART..............1 2<br>CAR/TRUCK ....1 2<br>BOAT WITH MOTOR ....1 2<br>BANANA BOAT...........................1 2	
15A	At any time in the past 12 months, has anyone sprayed the interior walls of your dwelling against mosquitoes?[2]	YES..1<br>NO...2<br>DON'T KNOW...8	<15D
15B	How many months ago was the house sprayed?[2]<br><br>IF LESS THAN ONE MONTH, RECORD '00' MONTHS AGO.	MONTHS AGO....................... |__|__|	
15C	Who sprayed the house?[2]	GOVERNMENT WORKER/PROGRAM ... 1<br>PRIVATE COMPANY............................... 2<br>HOUSEHOLD MEMBER.......................... 3<br><br>OTHER ________________________ 6<br>(SPECIFY)<br>DON'T KNOW... 8	
15D	At any time in the past 12 months, have the walls in your dwelling been plastered or painted?	YES..1<br>NO...2<br>DON'T KNOW...8	16

15E	How many months ago were the walls plastered or painted?<br><br>IF LESS THAN ONE MONTH, RECORD ‘00’ MONTHS AGO.	MONTHS AGO	
15F	Have any of the following been used in your living space over the last week:<br><br>Mosquito coils?<br><br>Insecticide spray (eg. DOOM, Rungu, Expel)?<br><br>Repellents?	YES NO<br>Mosquito coils..............................1 2<br><br>Insecticide spray..........................1 2<br><br>Repellents...................................1 2	
16	Does your household have any mosquito nets that can be used while sleeping?	YES .... 1<br>NO 2	→ 27
17	How many mosquito nets does your household have?<br><br>IF 7 OR MORE NETS, RECORD ‘7’.	NUMBER OF NETS.........................	
17a	Has anyone in your household ever sold or given away a mosquito net?	YES, SOLD A MOSQUITO NET .... 1<br>YES, GAVE AWAY A MOSQUITO NET... 2<br>NO .... 3<br>DON’T KNOW... 4<br>REFUSED 5	

[1] Categories to be developed locally and revised based on the pretest; however, the broad categories must be maintained. In some countries, it may be desirable to ask an additional question on the material of walls or ceilings.

[2] This question should be deleted in countries that do not have an indoor residual spraying program for mosquitoes.

18	ASK RESPONDENT TO SHOW YOU THE NET(S) IN THE HOUSEHOLD.<br><br>IF MORE THAN THREE NETS, USE ADDITIONAL QUESTIONNAIRE(S).	NET #1<br><br>OBSERVED .... 1<br>NOT<br>OBSERVED 2	NET #2<br><br>OBSERVED............. 1<br>NOT<br>OBSERVED............. 2	NET #3<br><br>OBSERVED .... 1<br>NOT<br>OBSERVED 2
19	How long ago did your household obtain the mosquito net?	MOS AGO |__|__| <br>MORE THAN 3 YEARS AGO.......................... 95	MOS AGO |__|__| <br>MORE THAN 3 YEARS AGO 95	MOS AGO |__|__| <br>MORE THAN 3 YEARS AGO 95
20a	OBSERVE OR ASK THE BRAND OF MOSQUITO NET.<br><br>IF BRAND IS UNKNOWN, AND YOU CANNOT OBSERVE THE NET, SHOW PICTURES OF TYPICAL NET TYPES/BRANDS TO RESPONDENT.	'PERMANENT' NET[1]<br>Permanet…………11<br>Olyset…………….12<br>MamaSafeNite…..13<br>NetProtect……….14<br><br>Other/Don't Know……16<br><br>'PRETREATED' NET[2]<br>ICONET………..21<br>Fennet………….22<br>KO Nets………..23<br>Safinet………...24<br><br>Other/Don't Know….. 26	'PERMANENT' NET[1]<br>Permanet………11<br>Olyset………….12<br>MamaSafeNite…13<br>NetProtect…….14<br><br>Other/Don't Know……16<br><br>'PRETREATED' NET[2]<br>ICONET………..21<br>Fennet………….22<br>KO Nets………..23<br>Safinet………...24<br><br>Other/Don't Know. 26	'PERMANENT' NET[1]<br>Permanet………11<br>Olyset………….12<br>MamaSafeNite..13<br>NetProtect……….14<br><br>Other/Don't Know……16<br><br>'PRETREATED' NET[2]<br>ICONET………..21<br>Fennet………….22<br>KO Nets………..23<br>Safinet………...24<br><br>Other/Don't Know….. 26

		OTHER……………….31<br>DON'T KNOW<br>BRAND……………..98	OTHER……………….31<br>DON'T KNOW<br>BRAND……………..98	OTHER……………….31<br>DON'T KNOW<br>BRAND……………..98
20b	Did you get the net through a mass distribution campaign, school distribution, during an antenatal care visit, or during an under five visit?<br><br>IF AT THE CLINIC FOR ANC OR UNDER FIVE VISIT, PROBE TO BE SURE IT SURE AT THE ANC OR UNDER FIVE CLINIC	YES, MASS DISTRIBUTION............ 1<br>YES, SCHOOL............ 2<br>YES, ANC………….. 3<br>YES, UNDER FIVE VISIT .... 4<br>NO 5	YES, MASS DISTRIBUTION............1<br>YES, SCHOOL ....2<br>YES, ANC………….. 3<br>YES, UNDER FIVE VISIT ....4<br>NO..............................5	YES, MASS DISTRIBUTION............ 1<br>YES, SCHOOL............ 2<br>YES, ANC………….. 3<br>YES, UNDER FIVE VISIT........................... 4<br>NO 5
20c	Where did you obtain the net?	GOVERNMENT CLINIC/HOSPITAL<br>NEIGHBORHOOD HEALTH COMMITTEE (NHC)<br>COMMUNITY HEALTH WORKER (CHW) / AGENT<br>RETAIL SHOP<br>PHARMACY<br>WORKPLACE<br>OTHER (SPECIFY)__________<br>DON'T KNOW	GOVERNMENT CLINIC/HOSPITAL<br>NEIGHBORHOOD HEALTH COMMITTEE (NHC)<br>COMMUNITY HEALTH WORKER (CHW) / AGENT<br>RETAIL SHOP<br>PHARMACY<br>WORKPLACE<br>OTHER (SPECIFY)_______<br>DON'T KNOW	GOVERNMENT CLINIC/HOSPITAL<br>NEIGHBORHOOD HEALTH COMMITTEE (NHC)<br>COMMUNITY HEALTH WORKER (CHW) / AGENT<br>RETAIL SHOP<br>PHARMACY<br>WORKPLACE<br>OTHER (SPECIFY)__________<br>DON'T KNOW
20d	Did you purchase the net?	YES…...........................1<br>NO.(skip to 21)…...........2	YES…........................1<br>NO.(skip to 21)…........2	YES…...........................1<br>NO.(skip to 21)…...........2

		NOT SURE…................8	NOT SURE…..............8	NOT SURE…................8
20e	How much did you pay for the net when it was purchased?	In Kwacha [ ][ ]	In Kwacha [ ][ ]	In Kwacha [ ][ ]
21	When you got the net, was it already factory-treated with an insecticide to kill or repel mosquitoes?	YES..............................1<br>NO................................2<br>NOT SURE...................8	YES .... 1<br>NO .... 2<br>NOT SURE 8	YES..............................1<br>NO................................2<br>NOT SURE...................8
22	Since you got the mosquito net, was it ever soaked or dipped in a liquid to kill or repel mosquitoes or bugs?	YES..............................1<br>NO................................2<br>(SKIP TO 24)<br>NOT SURE...................8	YES .... 1<br>NO .... 2<br>(SKIP TO 24)<br>NOT SURE 8	YES..............................1<br>NO................................2<br>(SKIP TO 24)<br>NOT SURE...................8
23	How long ago was the net last soaked or dipped?<br><br>IF LESS THAN 1 MONTH AGO, RECORD >00' MONTHS. IF LESS THAN 2 YEARS AGO, RECORD MONTHS AGO. IF '12 MONTHS AGO' OR '1 YEAR AGO,' PROBE FOR EXACT NUMBER OF MONTHS.	MOS AGO [ ][ ]<br>MORE THAN 2 YEARS AGO...........95<br>NOT SURE..............98	MOS AGO [ ][ ]<br>MORE THAN 2 YEARS AGO ....95<br>NOT SURE 98	MOS AGO [ ][ ]<br>MORE THAN 2 YEARS AGO...........95<br>NOT SURE 98
23a	Where was the net soaked or dipped?	HOME…………………1<br>GOVERNMENT CLINIC/HOSPITAL…..2<br>RETAIL SHOP………..3<br>PHARMACY………….4<br>WORKPLACE………..5<br>OTHER (SPECIFY)_______.....6<br>DON'T KNOW………..7	HOME<br>GOVERNMENT CLINIC/HOSPITAL<br>RETAIL SHOP<br>PHARMACY<br>WORKPLACE<br>OTHER (SPECIFY)_______<br>DON'T KNOW	HOME<br>GOVERNMENT CLINIC/HOSPITAL<br>RETAIL SHOP<br>PHARMACY<br>WORKPLACE<br>OTHER (SPECIFY)__________<br>DON'T KNOW
23b	Did you pay to soak or dip the net?	YES..............................1	YES...........................1	YES..............................1

		NO.(skip to 23d).............2<br><br>NOT SURE..................8	NO.(skip to 23d)...........2<br><br>NOT SURE...............8	NO.(skip to 23d).............2<br><br>NOT SURE..................8
23c	How much did you pay to soak or dip the net?	In Kwacha |__|__|__|	In Kwacha |__|__|__|	In Kwacha |__|__|__|
23d	PLEASE RECORD OR ASK THE GENERAL CONDITION OF THE NET.	1 Good (no holes)<br><br>2 Fair (no holes that fit a torch battery)<br><br>3 Poor (1-4 holes that fit a torch battery)<br><br>4 Unsafe (>5 Holes that fit a torch battery)<br><br>5 Unused (still in package)<br><br>98 Unknown	1 Good (no holes)<br><br>2 Fair (no holes that fit a torch battery)<br><br>3 Poor (1-4 holes that fit a torch battery)<br><br>4 Unsafe (>5 Holes that fit a torch battery)<br><br>5 Unused (still in package)<br><br>98 Unknown	1 Good (no holes)<br><br>2 Fair (no holes that fit a torch battery)<br><br>3 Poor (1-4 holes that fit a torch battery)<br><br>4 Unsafe (>5 Holes that fit a torch battery)<br><br>5 Unused (still in package)<br><br>98 Unknown
23e	PLEASE RECORD OR ASK THE COLOR OF THE NET.	1. Green<br>2. Blue<br>3. Red<br>4. White<br>5. Black<br>Other ________	1. Green<br>2. Blue<br>3. Red<br>4. White<br>5. Black<br>Other ________	1. Green<br>2. Blue<br>3. Red<br>4. White<br>5. Black<br>Other ________
23f	PLEASE RECORD OR ASK THE SHAPE OF THE NET.	1. Conical<br>2. Rectangular<br>3. Other ________	1. Conical<br>2. Rectangular<br>3. Other ________	1. Conical<br>2. Rectangular<br>3. Other ________
23g	In the last month, has the net gotten any new holes?	YES..............................1<br><br>NO.(skip to 24).............2<br><br>DON'T KNOW................8	YES ….............................1<br><br>NO.(skip to 24)............2<br><br>DON'T KNOW.............8	YES..............................1<br><br>NO.(skip to 24).............2<br><br>DON'T KNOW..............8
23h	What caused the new holes?	1 Tore or split when caught on object<br><br>2 Was burned	1 Tore or split when caught on object<br><br>2 Was burned	1 Tore or split when caught on object<br><br>2 Was burned

		3 Was caused by animals<br><br>4 Children<br><br>5 In another way (specify)<br>____________________<br><br>98 Don't Know	3 Was caused by animals<br><br>4 Children<br><br>5 In another way (specify)<br>____________________<br><br>98 Don't Know	3 Was caused by animals<br><br>4 Children<br><br>5 In another way (specify)<br>____________________<br><br>98 Don't Know
23i	Have you tried to repair the new holes?	YES..............................1<br><br>NO.(skip to 23k).............2<br><br>DON'T KNOW...............8	YES ....1<br><br>NO.(skip to 23k).............2<br><br>DON'T KNOW...............8	YES..............................1<br><br>NO.(skip to 23k).............2<br><br>DON'T KNOW...............8
23j	If yes, what did you use to repair the holes?<br><br>SKIP TO	1 Stitch<br><br>2 Know/tie<br><br>3 Patch<br><br>5 Other<br><br>98 Don't Know	1 Stitch<br><br>2 Know/tie<br><br>3 Patch<br><br>5 Other<br><br>98 Don't Know	1 Stitch<br><br>2 Know/tie<br><br>3 Patch<br><br>5 Other<br><br>98 Don't Know
23k	If no, what it the main reason you did not try to repair the holes?	1 Too busy<br><br>2 Not necessary<br><br>3 Don't know how to repair<br><br>5 Other<br><br>98 Don't Know	1 Too busy<br><br>2 Not necessary<br><br>3 Don't know how to repair<br><br>5 Other<br><br>98 Don't Know	1 Too busy<br><br>2 Not necessary<br><br>3 Don't know how to repair<br><br>5 Other<br><br>98 Don't Know
23l	Which of these statements best describes the net?<br><br>PLEASE ASK THE RESPONDENT.	1 Still in good condition<br><br>2 Net is beginning to fall apart and should be replaced soon<br><br>3 Net is no longer useable and needs to be replaced<br><br>98 Don't Know	1 Still in good condition<br><br>2 Net is beginning to fall apart and should be replaced soon<br><br>3 Net is no longer useable and needs to be replaced<br><br>98 Don't Know	1 Still in good condition<br><br>2 Net is beginning to fall apart and should be replaced soon<br><br>3 Net is no longer useable and needs to be replaced<br><br>98 Don't Know
23m	Is the net hanging for sleeping?<br><br>PLEASE OBSERVE OR ASK IF THE NET IS HANGING	YES..............................1	YES.............................1	YES..............................1

		NO..............................2	NO..............................2	NO..............................2
24	Did anyone sleep under this mosquito net last night?	YES.............................1<br><br>NO..............................2<br><br>(SKIP TO 25b) =——┤<br><br>NOT SURE...................8	YES .... 1<br><br>NO .... 2<br><br>(SKIP TO 25b) =——┤<br><br>NOT SURE 8	YES.............................1<br><br>NO..............................2<br><br>(SKIP TO 25b) =——┤<br><br>NOT SURE...................8

"Permanent" is a factory treated net that does not require any further treatment.

2 "Pretreated" is a net that has been pretreated, but requires further treatment after 6-12 months.

		NET #1	NET #2	NET #3
25a	Who slept under this mosquito net last night?<br><br>RECORD THE RESPECTIVE LINE NUMBER FROM THE HOUSEHOLD SCHEDULE.	NAME ___________<br>LINE NO [ ][ ]<br><br>NAME ___________<br>LINE NO [ ][ ]<br><br>NAME ___________<br>LINE NO [ ][ ]<br><br>NAME ___________<br>LINE NO [ ][ ]<br><br>NAME ___________<br>LINE NO [ ][ ]	NAME ___________<br>LINE NO [ ][ ]<br><br>NAME ___________<br>LINE NO [ ][ ]<br><br>NAME ___________<br>LINE NO [ ][ ]<br><br>NAME ___________<br>LINE NO [ ][ ]<br><br>NAME ___________<br>LINE NO [ ][ ]	NAME ___________<br>LINE NO [ ][ ]<br><br>NAME ___________<br>LINE NO [ ][ ]<br><br>NAME ___________<br>LINE NO [ ][ ]<br><br>NAME ___________<br>LINE NO [ ][ ]<br><br>NAME ___________<br>LINE NO [ ][ ]
25b	What is the <u>main</u> reason that nobody slept under this bed net last night?	NO MOSQUITOES .... 1<br>THERE IS NO MALARIA ...... 2<br>TOO HOT 3	NO MOSQUITOES.............. 1<br>THERE IS NO MALARIA...... 2<br>TOO HOT........................... 3	NO MOSQUITOES........... 1<br>THERE IS NO MALARIA ... 2<br>TOO HOT........................ 3

	**RECORD ONE ANSWER**	DON'T LIKE SMELL............4<br>FEEL 'CLOSED IN' ....5<br>NET TOO OLD OR TORN.....6<br>NET TOO DIRTY..................7<br>NET NOT AVAILABLE LAST<br>NIGHT (WASHING) ....8<br>USUAL USER DID NOT SLEEP<br>HERE LAST NIGHT ....9<br>NET WAS NOT NEEDED<br>LAST NIGHT ....10<br>NO PLACE TO HANG IT.... 11<br>OTHER (*specify*) ....96<br>DON'T KNOW 98	DON'T LIKE SMELL............4<br>FEEL 'CLOSED IN'..............5<br>NET TOO OLD OR TORN ....6<br>NET TOO DIRTY ....7<br>NET NOT AVAILABLE LAST<br>NIGHT (WASHING) ....8<br>USUAL USER DID NOT<br>SLEEP HERE LAST NIGHT ...9<br>NET WAS NOT NEEDED<br>LAST NIGHT.....................10<br>NO PLACE TO HANG IT... 11<br>OTHER (*specify*)..............96<br>DON'T KNOW 98	DON'T LIKE SMELL..........4<br>FEEL 'CLOSED IN'............5<br>NET TOO OLD OR TORN .6<br>NET TOO DIRTY ....7<br>NET NOT AVAILABLE LAST<br>NIGHT (WASHING) ....8<br>USUAL USER DID NOT<br>SLEEP HERE LAST NIGHT.9<br>NET WAS NOT NEEDED<br>LAST NIGHT..................10<br>NO PLACE TO HANG IT 11<br>OTHER (*specify*)............96<br>DON'T KNOW 98
26		GO BACK TO 18 FOR NEXT NET; OR, IF NO MORE NETS, GO TO 27.	GO BACK TO 18 FOR NEXT NET; OR, IF NO MORE NETS, GO TO 27.	GO BACK TO 18 IN THE FIRST COLUMN OF NEW QUESTIONNAIRE; OR, IF NO MORE NETS, GO TO 27.

LINE NO.	ECONOMIC BURDEN OF MALARIA										
	Was (NAME) sick with malaria during 2016/17 cropping season<br><br>IF NO SKIP TO NEXT PERSON,	Was (NAME) sick with malaria during Nov 2017 to May 2018<br><br>IF NO SKIP TO NEXT PERSON,	Number of times (NAME) has had Malaria in the Nov. 2017 to May 2018 crop season?	What were the main farm activities when (NAME) was sick?<br>1= Ploughing /hoeing and planting<br>2=weeding<br>3=harvesting<br>4=1+3<br>5=Others	No. of days (NAME) stopped completely work as a result of malaria during 2017/18 season? (Members >12 years)	No. of days (NAME) was partially at work to recover from disease (recovery period) or remain with fatigue and weakness during 2017/18 season?	By how much working capacity/effort of (NAME >=18 years) reduced compared to his/her normal health capacity/effort (%)	No. of work days lost by (NAME) because of taking care of malaria sick household members (caretaker)-this applies only for h/h member >=12 years	Where did (NAME) seek treatment/expert advice?<br>1=Village clinic;<br>2= hospital<br>3=traditional healer<br>4=did not seek treatment	After how many days of sickness did (NAME) go to hospital?	Total round trip transportation cost both for patient and caretaker (if any) (kwacha)
(1)	(9.1)	(9.2)	(9.3)	(9.4)	(9.5)	(9.6)	(9.7)	(9.8)	(9.9)	(9.10)	
	YES, NO DK	YES, NO DK									

LINE NO.	ECONOMIC BURDEN OF MALARIA <12 Years)

	Is (NAME) living with the biological father	Is (NAME) living with the biological mother	Is (NAME) currently attending formal school?<br>1=YES>><br>0=NO>>>	If **D5.1=No**, why not?<br>[and skip to next person]<br>(Use Code 3)	If **D5.1= Yes**, in which grade? Use Code 4)	Distance (walking minutes) to (NAME) school from home	How many hours does this person spend at school every day?	In the last 12 months, how much did you spend? School Fees*1 in kwacha	Did (NAME) miss school last month due to malaria related illness?	If yes days miss s the c
(1)	(9.1)	(9.2)	(9.3)	(9.4)	(9.5)	(9.6)	(9.7)	(9.8)	(9.9)	
	YES, NO DK	YES, NO DK							YES, NO DK	

**Code for E3**:	<u>**Code for E4:**</u>		<u>**Codes E10**</u>
1=Not yet schooling age<br>2=Can't pay school fee<br>3=No interest<br>4=Married/pregnant<br>5=Have jobs<br>6=Busy for HH work<br>7=Busy for own farm work<br>8=Choose to drop out for other reasons<br>9=long vacation<br>10=Disabled<br>11=School too far<br>12=Other (specify	0=Pre-Primary<br>1=Primary 1<br>2=Primary 2<br>3=Primary 3<br>4=Primary 4<br>5=Primary 5<br>6=Primary 6<br>7=Primary 7<br>8=Primary 8<br>9=Secondary 1 (OL1) or (Jr. I)<br>10=Secondary 2 (OL2) or (Jr. II)	11=Secondary 3 (OL3)<br>12=Secondary 4 (OL4)<br>13=Secondary 5 (AL1)<br>14=Secondary 6 (AL2)<br>15=Tertiary 1<br>16=Tertiary 2<br>17=Tertiary 3<br>18=Tertiary 4<br>19=Tertiary 5<br>20=Post graduate<br>99= Never in school	1=child was sick/ill<br>2=fees shortages<br>3=food shortage<br>4=Others (specify)…………..

*1 School fee is all payment made to school except transportation, uniform, and textbooks

HOUSE SCREENING												
Is your household aware of house screening (doors/windows/eaves) as a method for preventing malaria?<br><br>1=Yes>>**6A2**;<br>0=No>>6A12	Which year did your household hear about or become aware of house screening for the first time **(YYYY)**	How did you first learn about screening?<br><br>**Code A**	Does any window/door/eave of your main house have a screen<br><br>1=Yes>>**6A5**; 0=No<br>**enumerator to observe**	If windows are boarded up, glazed or screened, what primary material is used to do so?<br><br>**enumerator to observe**	What proportion of the windows of the main house are screened?<br>**(Enumerators to observe screens**	What proportion of the main house are screened?<br>**(Enumerators to observe screens**	What proportion of the eaves of the main house are screened?	When did your household put screening in windows/Doors/eaves **(YYYY)**<br><br>**6A9**	Maintenance of screens		How many households in your village do you know that are have put screens in the windows/doors/eaves of their main houses?	If you have screens, how effective is screening in malaria prevention?<br><br>**Code C**
									Do you do any maintenance on the screen in the doors/windows/eaves?<br><br>1=Yes>>**6A11**; 0=No>>A12	Will you put screens in windows/doors/eaves in future if you happen to build a new house?<br><br>**1=Yes**		

				Code B						0=No		
6A1	6A2	6A3	6A4	6A5	6A6	6A7	6A8		6A10	6A11	6A12	6A13

Codes A	Code B	Code C
1.= Govt health department<br>2= NGO/CBO<br>3=friend/neighbour<br>4= Other health research centre<br>5= Fellow farmer<br>6= Radio<br>7=TV<br>8=others	1=wood boards<br>2=glass<br>3=metal netting<br>4=fabric netting<br>5=plastic netting<br>6=other	1=very effective<br>2=somehow effective<br>3 =not effective<br>4=Not applicable
